# The NAD^+^ Precursor Nicotinamide Riboside Rescues Mitochondrial Defects and Neuronal Loss in iPSC derived Cortical Organoid of Alpers' Disease

**DOI:** 10.7150/ijbs.91624

**Published:** 2024-01-25

**Authors:** Yu Hong, Zhuoyuan Zhang, Tsering Yangzom, Anbin Chen, Bjørn Christian Lundberg, Evandro Fei Fang, Richard Siller, Gareth John Sullivan, Jiri Zeman, Charalampos Tzoulis, Laurence A. Bindoff, Kristina Xiao Liang

**Affiliations:** 1 Department of Clinical Medicine (K1), University of Bergen, Bergen, Norway.; 2 Neuro-SysMed, Center of Excellence for Clinical Research in Neurological Diseases, Haukeland University Hospital, Bergen, Norway.; 3 Department of Neurology, Beijing Tongren Hospital, Capital Medical University, Beijing, China.; 4 State Key Laboratory of Oral Diseases, National Clinical Research Center for Oral Diseases, West China School of Stomatology, Sichuan University, Chengdu, China.; 5 Department of Head and Neck Cancer Surgery, West China Hospital of Stomatology, Sichuan University, Chengdu, China.; 6 Centre for International Health, University of Bergen, Bergen, Norway.; 7 Department of Clinical Molecular Biology, Akershus University Hospital, University of Oslo, Oslo, Norway.; 8 The Norwegian Centre on Healthy Ageing, Oslo, Norway.; 9 Norwegian Center for Stem Cell Research, University of Oslo, 0317, Oslo, Norway.; 10 Department of Molecular Medicine, Institute of Basic Medical Sciences, University of Oslo, 0317, Oslo, Norway.; 11 Institute of Immunology, Oslo University Hospital, Oslo, Norway.; 12 Department of Pediatric Research, Oslo University Hospital, Oslo, Norway.; 13 Department of Paediatrics and Inherited Metabolic Disorders, First Faculty of Medicine, Charles University, Prague, Czech Republic.; 14 KG Jebsen Center for Parkinson's disease, University of Bergen, Bergen, Norway.; 15 Department of Neurology, Haukeland University Hospital, Bergen, Norway.; 16 National Advisory Unit for Congenital Metabolic Diseases, Oslo University Hospital, Oslo, Norway.

**Keywords:** Alpers' disease, induced pluripotent stem cells, cortical organoids, mitochondrial function, NAD^+^, NR

## Abstract

Alpers' syndrome is an early-onset neurodegenerative disorder usually caused by biallelic pathogenic variants in the gene encoding the catalytic subunit of polymerase-gamma (POLG), which is essential for mitochondrial DNA (mtDNA) replication. The disease is progressive, incurable, and inevitably it leads to death from drug-resistant status epilepticus. The neurological features of Alpers' syndrome are intractable epilepsy and developmental regression, with no effective treatment; the underlying mechanisms are still elusive, partially due to lack of good experimental models. Here, we generated the patient derived induced pluripotent stem cells (iPSCs) from one Alpers' patient carrying the compound heterozygous mutations of A467T (c.1399G>A) and P589L (c.1766C>T), and further differentiated them into cortical organoids and neural stem cells (NSCs) for mechanistic studies of neural dysfunction in Alpers' syndrome. Patient cortical organoids exhibited a phenotype that faithfully replicated the molecular changes found in patient postmortem brain tissue, as evidenced by cortical neuronal loss and depletion of mtDNA and complex I (CI). Patient NSCs showed mitochondrial dysfunction leading to ROS overproduction and downregulation of the NADH pathway. More importantly, the NAD^+^ precursor nicotinamide riboside (NR) significantly ameliorated mitochondrial defects in patient brain organoids. Our findings demonstrate that the iPSC model and brain organoids are good *in vitro* models of Alpers' disease; this first-in-its-kind stem cell platform for Alpers' syndrome enables therapeutic exploration and has identified NR as a viable drug candidate for Alpers' disease and, potentially, other mitochondrial diseases with similar causes.

## Introduction

Alpers' syndrome, a devastating early-onset neurodegenerative disorder is tightly linked to abnormalities in the function of mitochondria and mutations in the catalytic subunit of polymerase gamma (*POLG*) gene 1. The syndrome, which typically follows an autosomal mode of inheritance, manifests a range of debilitating symptoms including intractable seizures, developmental regression, hypotonia, ataxia, and liver failure. In some severe instances, it can lead to premature death 2. Symptoms usually emerge between first and third years of life, with the disorder estimated to affect 1 in 100,000 newborns 3.

Unfortunately, Alpers' syndrome's pathogenesis remains obscure, making the development of direct treatment modalities challenging. Consequently, current interventions focus on symptomatic relief and providing supportive care. Certain drugs previously utilized, like valproic acid, are now largely discarded due to their severe hepatotoxic effects and persistence of drug-resistant epilepsy 4, 5. This underlines the crucial need for continuing research into the root cause of Alpers' syndrome, which would pave the way for devising strategies to limit disease progression and generate efficacious treatments.

The *POLG* gene, situated on the long arm of chromosome 15, encodes for polymerase γ, an enzyme chiefly responsible for replication and repair of mitochondrial DNA (mtDNA) 1. More than 90% of Alpers' syndrome cases are linked to mutations in the* POLG* gene. These mutations are typically inherited in an autosomal recessive manner, often from parents who are asymptomatic carriers. The mutations commonly observed include homozygous p.A467T or p.W748S transitions and heterozygous p.T851A and p.R1047W mutations, all of which impact the function and phenotypic manifestation of polymerase γ (Pol γ) A gradual deficiency in Pol γ contributes directly to mtDNA depletion, instigating serious harm to the mitochondrial respiratory chain, particularly leading to the breakdown of CI 6, 7. The ensuing severe mitochondrial dysfunction manifests as ataxia, hepatic failure, and even premature death. Since Alpers' syndrome is a metabolic disorder, deepening our understanding of its pathogenesis could pave the way for a more comprehensive grasp of mitochondrial metabolism. This could potentially open new avenues for more effective treatments.

Unraveling the pathogenesis of neurodegenerative diseases, including Alpers' disease, is a complex task due largely to the intricacy of the human nervous system. To date, most research efforts have relied on genetically modified cell lines that is limited to relatively single immortalized cell lines 8 or rodent primary neural stem cells (NSCs) 9. However, these models may not adequately represent the multifaceted *in vivo* microenvironment of the human body. *In vivo* studies using animal models provide a simplified, cost-effective approach to neurodegeneration research. Nevertheless, their applicability is constrained due to inherent differences between human and animal models 10. Furthermore, neuroprotective drugs showing promise in animal models often disappoint when tested in human clinical trials. Given these limitations, there is an urgent need for a more accurate disease model that truly emulates the complexities of neurodegenerative diseases in humans. Such a model could unlock insights into disease mechanisms and expedite the development of effective therapeutic strategies.

Introduced by Shinya Yamanaka in 2007, human induced pluripotent stem cells (iPSCs), have emerged as a promising source of cells for in vitro studies and clinical applications in humans and patients 11. This innovative approach reverts human somatic cells, such as skin fibroblasts or peripheral blood mononuclear cells, back to a pluripotent state using various combinations of transcription factors 12. iPSCs derived from specific diseases can retain the patient's individual genetic mutations and avoid potential interspecies variation, offering a tremendous tool to study neurodegenerative diseases at the cellular level 13. To date, patient-specific iPSCs have been extensively utilized in the creation of diverse neuronal models 14 and in the exploration of mitochondrial diseases caused by mtDNA mutations, such as *Mitochondrial Encephalomyopathy*, *Lactic Acidosis*, and *Stroke-like Episodes (MELAS) syndrome* 15, 16, *Myoclonic Epilepsy with Ragged-Red Fibers (MERRF) syndrome* 17 and *Pearson syndrome* (PS) 18. However, most cellular assays primarily rely on traditional two-dimensional (2D) culture systems, which are increasingly recognized as inadequate for mimicking complex natural environments. Recent evidence supports the idea that three-dimensional (3D) cell culture systems, such as brain organoids derived from iPSCs, could address this limitation effectively. These 3D systems accurately reflect *in vivo* microenvironment 19, 20, providing robust models to emulate the disease profiles of neurodegenerative disease and conduct drug testing.

Nicotinamide adenine dinucleotide in its oxidized form (NAD^+^) is a vital molecule for life and health, playing a particularly crucial role in neuroprotection. NAD^+^'s fundamental molecular functions extend beyond its roles in energy metabolism and production, including glycolysis, β-oxidation, the TCA cycle, and oxidative phosphorylation (OXPHOS). It also contributes to cellular repair and resilience, factors that underpin its ability to protect the brain 21, 22. Research conducted on postmortem brain tissues, iPSC-derived cells, and animal models indicates a decline in NAD^+^ levels in the aging brain, as well as in prevalent neurodegenerative diseases, such as Alzheimer's disease (AD), Parkinson's disease (PD), Amyotrophic lateral sclerosis (ALS), and Huntington's disease (HTT) 23. The belief that the reduction in NAD^+^ is a primary cause, rather than a simple correlate, is bolstered by evidence showing that NAD^+^ augmentation-achieved through supplementation with NAD^+^ precursors like as nicotinamide riboside (NR) and nicotinamide mononucleotide (NMN), can alleviate disease pathologies in laboratory models of these diseases 23. Notably, clinical studies have demonstrated that NR is not only safe (up to 2 g/day for up to 3 months) and bioavailable, but it can also ameliorate some syndromes in patients with PD 24 and ALS 25.

To date, there has been a few reported uses of the iPSCs model for the study of Alpers' syndrome resulting from *POLG* mutation, and focused only on iPSCs and derived 2D neurons 26, 27. In this study, we established an iPSCs model of Alpers' syndrome, generated from the skin fibroblasts of one Alpers' patient. These cells were further differentiated into NSCs and 3D cortical to investigate the varied manifestations and pathogenesis of Alpers' disease. Both the iPSCs and NSCs derived from Alpers' patients exhibited some degree of mitochondrial dysfunction, the dysfunction being more pronounced in NSCs.

With the use of a cortical organoid model derived from iPSCs, we found that the mitochondrial alterations in the cortical organoids from Alpers' patient mirrored those observed in 2D NSCs. Additionally, a transcriptomic analysis revealed a downregulation of the NADH pathway and reduced expression of mitochondrial transcripts in both reprogrammed iPSCs and induced NSCs. This provided key insights into potential therapeutic approaches. Notably, subsequent studies in the cortical organoid model demonstrated that NR significantly rectified the structural disruptions and mitochondrial defects in brain organoids generated from an Alpers' syndrome patient. For the first time, we have constructed a stem cell model for Alpers' syndrome, paving the way for precise mechanistic studies and the exploration of potential drug development for this currently incurable disease.

## Results

### Alpers's iPSCs manifested a mild impairment of mitochondrial function compared to controls

**Figure 1:**
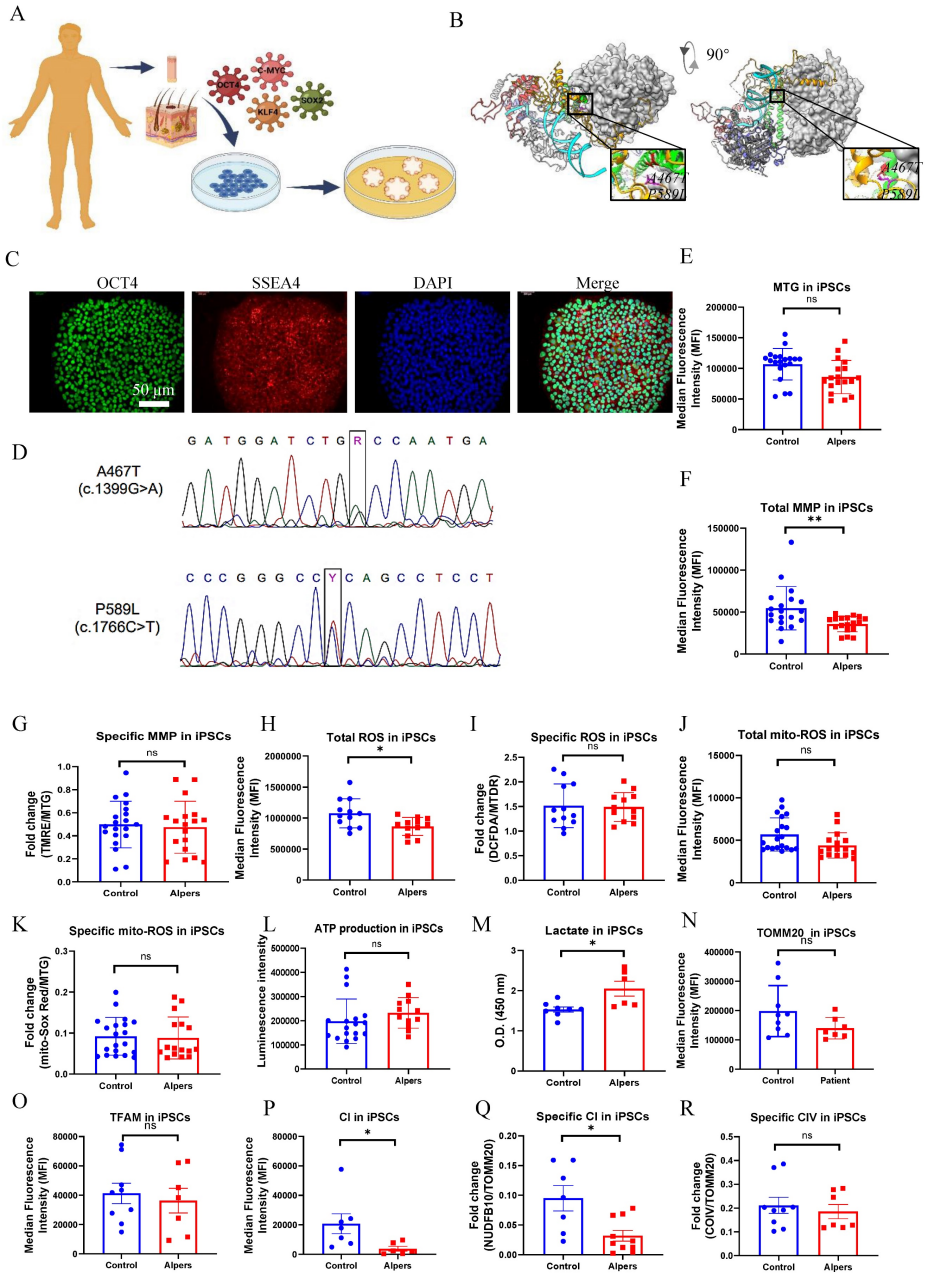
** Measurement of mitochondrial function, mtDNA alteration and mitochondrial complexes in Alpers' iPSCs.** A. Illustration of reprogramming of Alpers' patient's fibroblasts into iPSCs. B. Molecular Structure of POLG protein and position of A467T and P589L mutations. C. Representative fluorescent images of Alpers' iPSCs for pluripotent markers OCT4 and SSEA4. Nuclei are stained with DAPI (blue). Scale bar is 50 µm. D. A467T and P589L mutation verified by sanger sequencing using Alpers' reprogrammed iPSCs. E. Flow cytometric analysis for mitochondrial volume using MTG in iPSCs. F-G. Flow cytometric analysis for total MMP and specific MMP in iPSCs. H-K. Flow cytometric analysis for total ROS, specific ROS, total mito-ROS, and specific mito-ROS in iPSCs. L-M. Flow cytometric analysis for ATP and lactate production in iPSCs. N. Flow cytometric analysis for TOMM20 levels in iPSCs. O. Flow cytometric analysis for TFAM in iPSCs. P-R. Flow cytometric analysis for CI, specific CI, and specific CIV levels in iPSCs. The numbers of clones and replications in each experiment was listed in Table S 13.

We generated iPSCs from a patient with Alpers' syndrome (Figure 1A). The clinical manifestations of the patients were characterized by recurrent seizure, psychomotor regression and liver dysfunction. Gene mutation analysis showed that the patient carried heterozygous mutations A467T (c.1399G>A) and P589L (c.1766C>T). *POLG* gene mutations are located in the linker region that is in close proximity to the auxiliary subunit (Figure 1B). To validate the pluripotency and cellular purity of the iPSCs, we utilized flow cytometry, which confirmed that over 97% of the iPSCs derived from Alpers' patient expressed the pluripotent marker NANOG (Supplemental Figure 1). Fluorescent staining further confirmed that the iPSC colonies expressed the pluripotent markers OCT4 and SSEA4 (Figure 1C). Sanger sequencing analysis corroborated that two mutations present in the iPSCs were consistent with those identified in the patient (Figure 1D). We utilized five individual clones from three control lines, three clones derived from control Detroit 551 fibroblasts, one clone from CRL2097 fibroblasts and one clone from AG AG05836, as disease-free controls. We observed that the iPSCs derived from Alpers' patient exhibited typical colony morphology, similar to the control iPSCs (Supplemental Figure 2). To evaluate pluripotency and tri-lineage differentiation, we quantified the expression of relevant genes via RNA sequencing analysis. We found positive expressions of the pluripotent markers *NANOG* and *KLF4*; endoderm markers *CXCR4* and *GATA4*; mesoderm markers *TBX6*, *MSX1*, *OTX2*, *MESP1*, *SOX2*, and *MIXL1*; and ectoderm markers *NCAM1*, *TBX6*, *MSX1*, *OTX2*, *MESP1*, and *SOX2*. These markers were expressed in both the control line (Supplementary Figure 3) and the Alpers line (Supplementary Figure 4).

To determine if there were changes in mitochondrial function in iPSCs reprogrammed from Alpers' patient fibroblasts, we assessed mitochondrial membrane potential (MMP), mitochondrial mass, ROS levels and intracellular energy production. Mitochondrial mass and MMP were measured. Each was measured by flow cytometry after double-staining cells with MitoTracker Green (MTG) and tetramethylrhodamine ethyl ester (TMRE). To gauge MMP levels per mitochondrion, we measure the ratio between total MMPs and MTG, providing relative measurements of specific MMPs.

Our results showed that mitochondrial volume measured by MTG (Figure 1E) and total MMPs assessed by TMRE (Figure 1F) were reduced in Alpers' iPSCs compared to the control iPSCs. However, specific MMPs did not differ statistically (Figure 1G). ROS production was analyzed using 2', 7'-dichlorodihydrofluorescein diacetate (DCFDA) and MitoTracker Deep Red (MTDR) double staining and flow cytometry. Alpers' iPSCs produced less total ROS than the control iPSCs (Figure 1H). After normalizing total ROS by measuring mitochondrial mass MTDR, no difference was observed in ROS production per mitochondria (Figure 1I). Furthermore, total mitochondrial ROS measured by a mitochondrial ROS (mito-ROS)-sensitive fluorescent dye, did not differ between Alpers' and control iPSCs (Figure 1J). When normalized by MTG, specific mito-ROS levels remained statistically indistinct (Figure 1K).

ATP production per cell was assessed using a luminescence assay, revealing no difference in ATP levels between Alpers' and control iPSCs (Figure 1L). Moreover, to explore glycolysis activity in Alpers' iPSCs, we measured L-lactate levels and found them to be higher in Alpers' iPSCs compared to the control group (Figure 1M).

To further investigate the mtDNA copy number, we employed flow cytometry to indirectly mtDNA copy number by examining the levels of mitochondrial transcription factor A (TFAM) and mtDNA, both of which are bound together in molar amounts. We also sought to determine the mtDNA copy number per mitochondria by performing double staining with the mitochondrial import receptor subunit TOM20 (TOMM20) and then calculating the ratio of TFAM to TOMM20. Our findings revealed no significant difference in the expression of TFAM and TOMM20 proteins between the patient and control (Figure 1N, O).

Given the known association of oxidative phosphorylation (OXPHOS) complex deficiency with *POLG* mutations 28, we evaluated whether this also the case in iPSCs derived from Alpers' patient. We did this by using antibodies against the CI subunit NDUFB10 for measurement of CI level, the complex IV subunit COXIV to assess mitochondrial complex IV (CIV) level, and TOMM20 to quantify the mitochondrial volume. Following this, we applied flow cytometry. Our findings revealed that both total levels of NDUFB10 (Figure 1P) and specific levels of NDUFB10 when normalized to TOMM20 levels (Figure 1Q) were significantly reduced in Alpers' iPSCs. We did not find any significant changes in the total (Supplemental Figure 5) and specific CIV (Figure 1R) or the total TOMM20 level (Figure 1N) in Alpers' iPSCs compared to control iPSCs.

Collectively, these results suggest that we can successfully generate iPSCs from a patient with Alpers' syndrome and that the iPSCs derived from the patient exhibit mild mitochondrial alterations, including an elevated L-lactate level and a depletion of CI.

### Alpers' NSCs showed more severe impairment of mitochondrial function compared to controls

**Figure 2:**
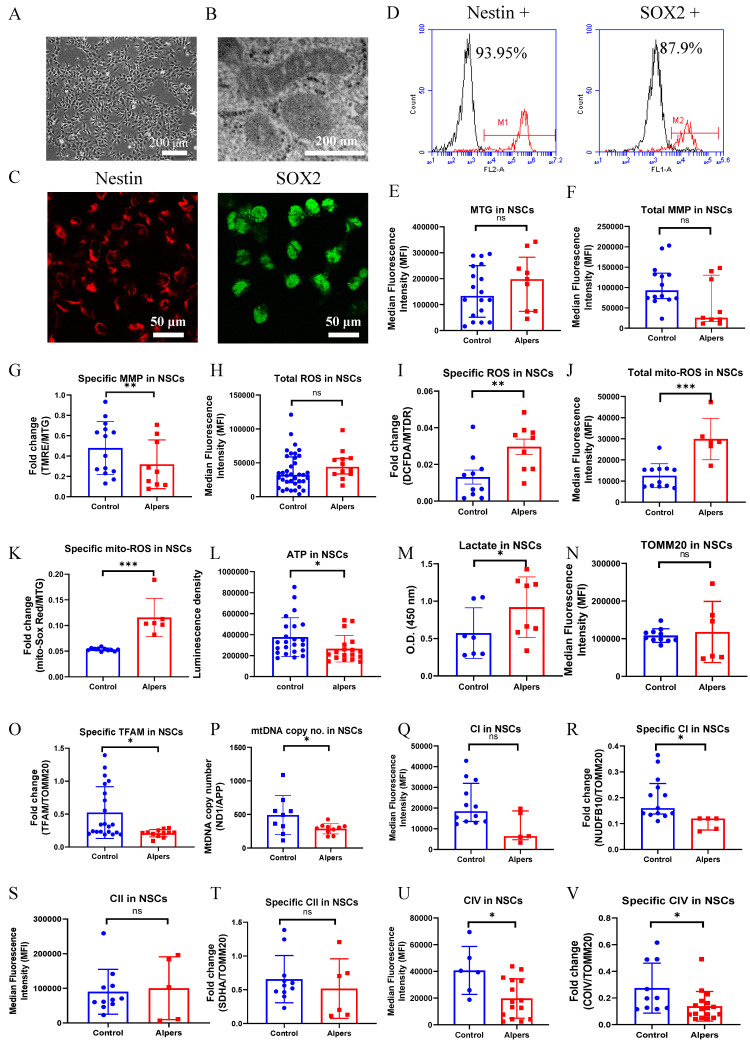
** Measurement of mitochondrial function, mtDNA alteration and mitochondrial complexes in Alpers' NSCs.** A. Representative phase-contrast images of NSCs of Alpers' patient. Scale bar is 200 µm. B. Representative TEM images of NSCs of Alpers' patient. Scale bar is 200 nm. C. Representative fluorescent images of Alpers' NSCs for NSC markers Nestin and SOX2. D. Flow cytometric analysis for NSC markers Nestin and SOX2 in NSCs. E. Flow cytometric analysis for mitochondrial volume using MTG in NSCs. F-G. Flow cytometric analysis for total MMP and specific MMP in NSCs. H-K. Flow cytometric analysis for total ROS, specific ROS, total mito-ROS, and specific mito-ROS in NSCs. L-M. Flow cytometric analysis for ATP and lactate production in NSCs. N. Flow cytometric analysis for TOMM20 levels in NSCs. O-P. Flow cytometric analysis for TFAM and mtDNA copy number levels in NSCs. Q-V. Flow cytometric analysis for CI, specific CI, CII, specific II, CIV, specific CIV levels in NSCs. The numbers of clones and replications in each experiment was listed in Table S 13.

Continuing our investigations, we utilized a method from previous research to further differentiate iPSCs into NSCs, and then conducted the same experiments as before to evaluate alterations in mitochondrial function in these NSCs. Alpers' NSCs were observed to display a conventional neural stem cell morphology (Figure 2A) and an unaltered mitochondrial double cristae morphology (Figure 2B). The NSCs, derived from iPSCs, showed the positive expression of neural stem cell markers, Nestin and SOX2 (Figure 2C). Utilizing flow cytometry, it was established that over 93% and 87% of Alpers' NSCs expressed Nestin and SOX2, respectively (Figure 2D).

Using the same TMRE/MTG double staining method as we did for iPSCs, it was found that specific MMPs were decreased in Alpers' NSCs compared to controls (Figure 2G). However, no significant variation was observed in total MMPs (Figure 2F) and mitochondrial volume, as indicated by MTG (Figure 2E). For Alpers' NSCs, a significant increase was observed in specific intracellular ROS (DCFDA/MTDR) and mitochondrial ROS at both total (MitoSOX Red) and specific level (MitoSOX Red/MTG) when compared to control NSCs (Figure 2I, J, K), yet the total intracellular ROS (DCFDA/MTDR) showed no difference (Figure 2H). A direct measurement of intracellular ATP production revealed a marked decrease in ATP production in Alpers' NSCs compared to the control NSCs (Figure 2L), whereas the L-lactate levels were found to be higher in Alpers' NSCs than in controls (Figure 2M).

In addition, we utilized indirect methods TFAM measurement and qPCR to evaluate the mtDNA levels of ND1/APP. Using flow cytometry, we discerned a marked decline in specific TFAM levels (TFAM/TOMM20) in Alpers' NSCs compared to controls (Figure 2O). Interestingly, we didn't observe any significant alterations in the levels of TOMM20 (Figure 2N). To corroborate the reduction in the mtDNA levels as observed through the ND1/APP measurements in Alpers' NSCs, we performed qPCR (Figure 2P), which confirmed the same. Further assessments of OXPHOS complexes I, II, and IV using flow cytometry revealed significantly decreased expression, both in terms of the total level and specificity of CI (Figure 2Q, R) and CIV (Figure 2U, V) in Alpers' NSCs compared to control NSCs. However, we did not notice any significant changes in the total (Figure 2S) and specific level of complex II (CII) (Figure 2T).

This body of evidence collectively suggests that NSCs in Alpers' syndrome are distinguished by profound mtDNA depletion and resultant mitochondrial dysfunction.

### Mitochondrial-related pathways were downregulated in Alpers' derived iPSCs and NSCs, and more pronounced in NSCs

**Figure 3:**
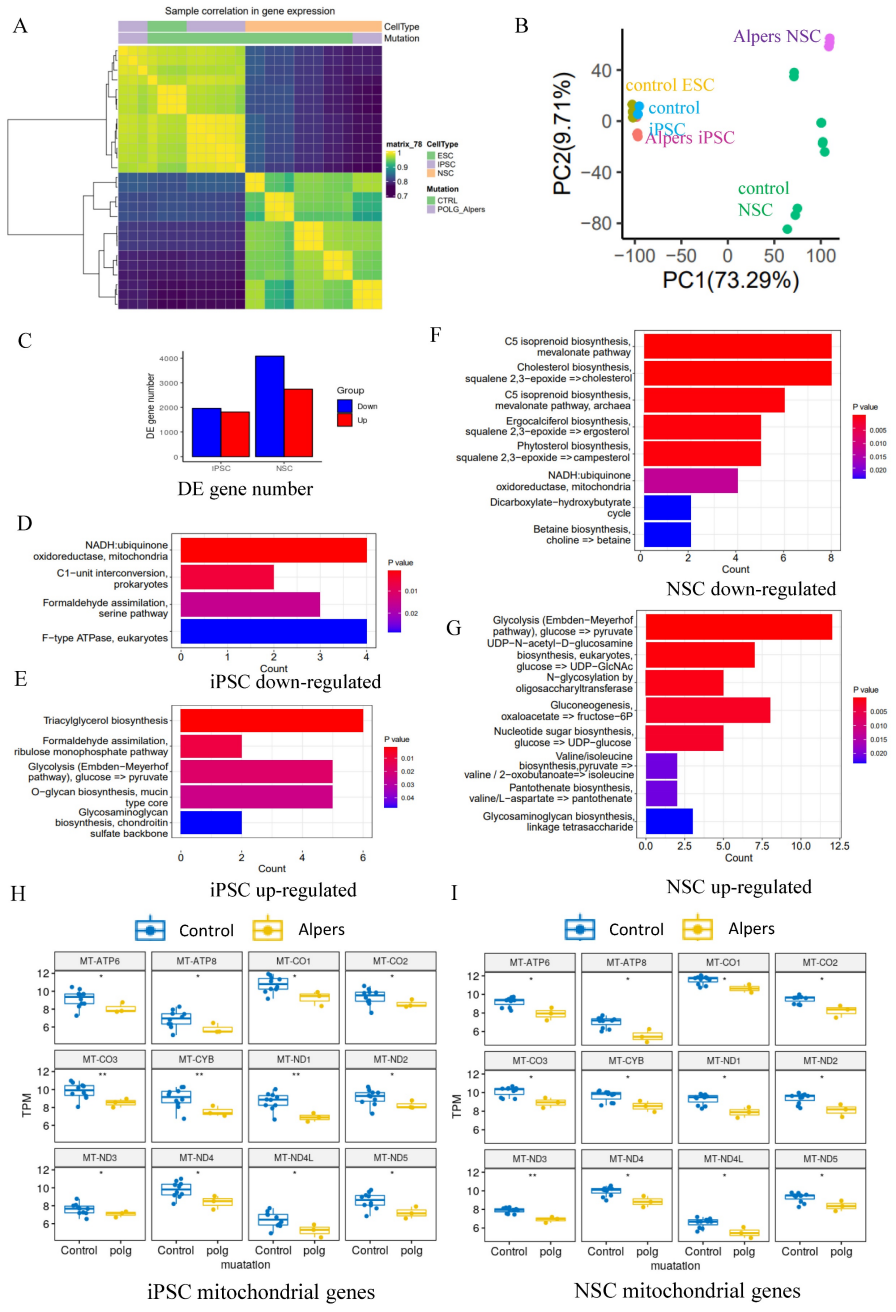
** Transcriptomic status and dysregulated mitochondrial related pathways of Alpers' patient derived iPSCs and NSCs.** A. Sample correlation in transcriptomic status of Alpers' patient derived iPSCs and NSCs, and the corresponding controls. B. PCA analysis of transcriptomic expression data for Alpers' patient derived iPSC and NSCs and controls. C. Number of upregulated and down-regulated DE genes between Alpers' and NSCs in iPSCs and NSCs, respectively. D. Downregulated metabolic pathways in Alpers' iPSCs compared with controls. E. Upregulated metabolic pathways in Alpers' iPSCs compared with controls. F. Downregulated metabolic pathways in Alpers' NSCs compared with controls. G. Upregulated metabolic pathways in Alpers' NSCs compared with controls. H-I. RNA expressions of mitochondrial genes in Alpers' iPSCs and NSCs.

To delve deeper into the comparison of transcriptome and gene alterations between Alpers' and control lines, we executed a transcriptome-wide RNA sequencing (RNA-seq) analysis on both iPSCs and induced NSCs derived from Alpers' and controls. Hierarchical clustering based on RNA transcripts displayed unique, independent clusters for NSCs and iPSCs (Figure 3A). Principal component analysis (PCA) authenticated the separation between NSCs and iPSCs, with the difference between Alpers' NSCs and control NSCs being more conspicuous than those between iPSCs (Figure 3B). The differential expression analysis unveiled that 1805 genes were upregulated, and 1905 genes were downregulated in Alpers' iPSCs compared with the control iPSCs (Figure 3C).

Relative to controls, Alpers' NSCs displayed 2725 upregulated genes and 4059 downregulated genes (Figure 3C). KEGG pathway analysis based on DEGs disclosed a downregulation of the mitochondrial NADH: ubiquinone oxidoreductase pathway in both Alpers' derived iPSCs (Figure 3D, E, and Table S1) and NSCs (Figure 3F and Table S2) compared to control cells. Interestingly, gluconeogenesis and glycolysis pathways were markedly upregulated in Alpers' NSCs (Figure 3G and Table S2), indicating that glycolysis might be compensating for energy production in Alpers' NSCs, a phenomenon providing indirect evidence of mitochondrial dysfunction. Moreover, Alpers' iPSCs and NSCs exhibited significantly lower levels of mitochondrial transcripts, encompassing *ATP6*,* ATP8*,* CO1*, *CO2*,* CO3*,* CYB*,* ND1*,* ND2*,* ND3*,* ND4*,* ND4L*, and* ND5*, relative to controls (Figure 3H, I). This observation aligns with the previously demonstrated mtDNA depletion in NSCs derived from Alpers' patients (Figure 2O, P).

Our finding showed an increase in ROS production in Alpers' NSCs (Figure 2K). Given that the balance between ROS production and the cellular antioxidant defense system is vital for maintaining redox homeostasis, and any disruption in this balance can lead to oxidative stress, we further assessed the antioxidant capacity of NSCs using the scRNA-seq analysis. We found that the expression of several key antioxidant genes was significantly altered, which indicates a response to the increased ROS production in Alpers' NSCs (Supplementary Figure 6A). Notably, the expression of genes such as *CAT*, *GPX1*, *GPX4*, *SOD1*, and *TXNRD1* was downregulated, as suggested by their negative log2 fold-change values. This downregulation implies a compromised cellular antioxidant defense capacity, which could exacerbate oxidative stress due to the inability to effectively neutralize the increased ROS levels. However, other genes, including *SOD2* and *PRDX5*, showed a significant upregulation, indicating that while some components of the antioxidant system are suppressed, others are activated, possibly as a compensatory mechanism to mitigate oxidative damage. The upregulation of *SOD2* is particularly relevant as it encodes mitochondrial superoxide dismutase, which suggests an enhanced defense against mitochondrial ROS. The gene *GPX3* showed the most significant downregulation, which implies a substantial decrease in the corresponding glutathione peroxidase activity, an enzyme critical for detoxifying peroxides. The dramatic decrease in GPX3 expression may be a key factor in the increased vulnerability to oxidative stress observed in Alpers' NSCs. Interestingly, genes such as *HMOX1* and *NQO1*, which are involved in the response to oxidative stress and are regulated by the transcription factor NRF2, were upregulated. This suggests that the NRF2-mediated oxidative stress response pathway may be activated in these cells.

Given our findings on the significant reductions in mitochondrial CI, III, IV protein levels, and mtDNA transcripts in Alpers' NSCs (Figure 2O-V) and that these could act as potential precursors to cellular stress leading to apoptosis, we further compared the anti- and pro-apoptotic genes as well as stress response pathway genes Alpers' NSCs versus controls. We found significant changes in the expression of several key apoptotic and stress response genes (Supplementary Figure 6B). *CASP3*, *CASP9*, and *CYCS*, which are key players in the apoptotic process, showed decreased expression as indicated by the negative log2 fold-change values. This downregulation suggests a reduced activation of apoptosis through both intrinsic mitochondrial pathways (as seen with *CASP9* and *CYCS*) and the execution phase (as noted with *CASP3*). On the other hand, anti-apoptotic genes such as *BCL2*, *BCL2L1*, and *MCL1* exhibited increased expression, suggesting a potential cellular response to mitigate the induction of apoptosis. The upregulation of these genes might be a compensatory mechanism to counteract mitochondrial dysfunction and maintain cellular viability. The *BAX* gene, a pro-apoptotic member, is also downregulated, which aligns with the observed trend of reduced pro-apoptotic activity. Surprisingly, *BID*, another pro-apoptotic gene, showed increased expression, which could suggest a complex and nuanced response to the mitochondrial dysfunction. Additionally, the activation of stress response pathways is evident from the increased expression of genes such as *ATF4* and *DDIT3*, which are involved in the endoplasmic reticulum stress response. The upregulation of these genes indicates that the cells may be undergoing stress likely related to the mitochondrial dysfunction observed. In addition, the enrichment in the upregulated DEGs further demonstrated the upregulation of gene expressions in crucial apoptotic pathways within Alpers' NSCs (Supplementary Figure 6C). Particularly, the intrinsic apoptotic signaling pathway, as a reaction to endoplasmic reticulum stress, and its negative regulation pathway are profoundly enriched, which may reflect an intensified stress response and potential dysregulation in homeostatic mechanisms. The regulatory pathways of both intrinsic and extrinsic apoptosis show significant changes, indicative of the complex nature of apoptotic dysregulation in Alpers' disease. The observable gene upregulations within the oxidative stress-induced intrinsic apoptotic signaling pathway included the key regulators such as *NOXA*, *BAK1*, *BAX*, *PUMA*, *APAF1*, *TRAF2*, and *UQCRH* (Supplementary Figure 6D). Taken together, this body of evidence proposes that mitochondrial-associated pathways are downregulated in both iPSCs and NSCs derived from Alpers' patient, with these alterations being more accentuated in NSCs. Additionally, altered expression of antioxidant and apoptotic genes indicates an adaptive response to increased ROS production and potential compensatory mechanisms to mitigate apoptosis and maintain cellular viability.

### Alpers' cortical organoids demonstrated cortical neuronal loss and astrocyte accumulation

**Figure 4:**
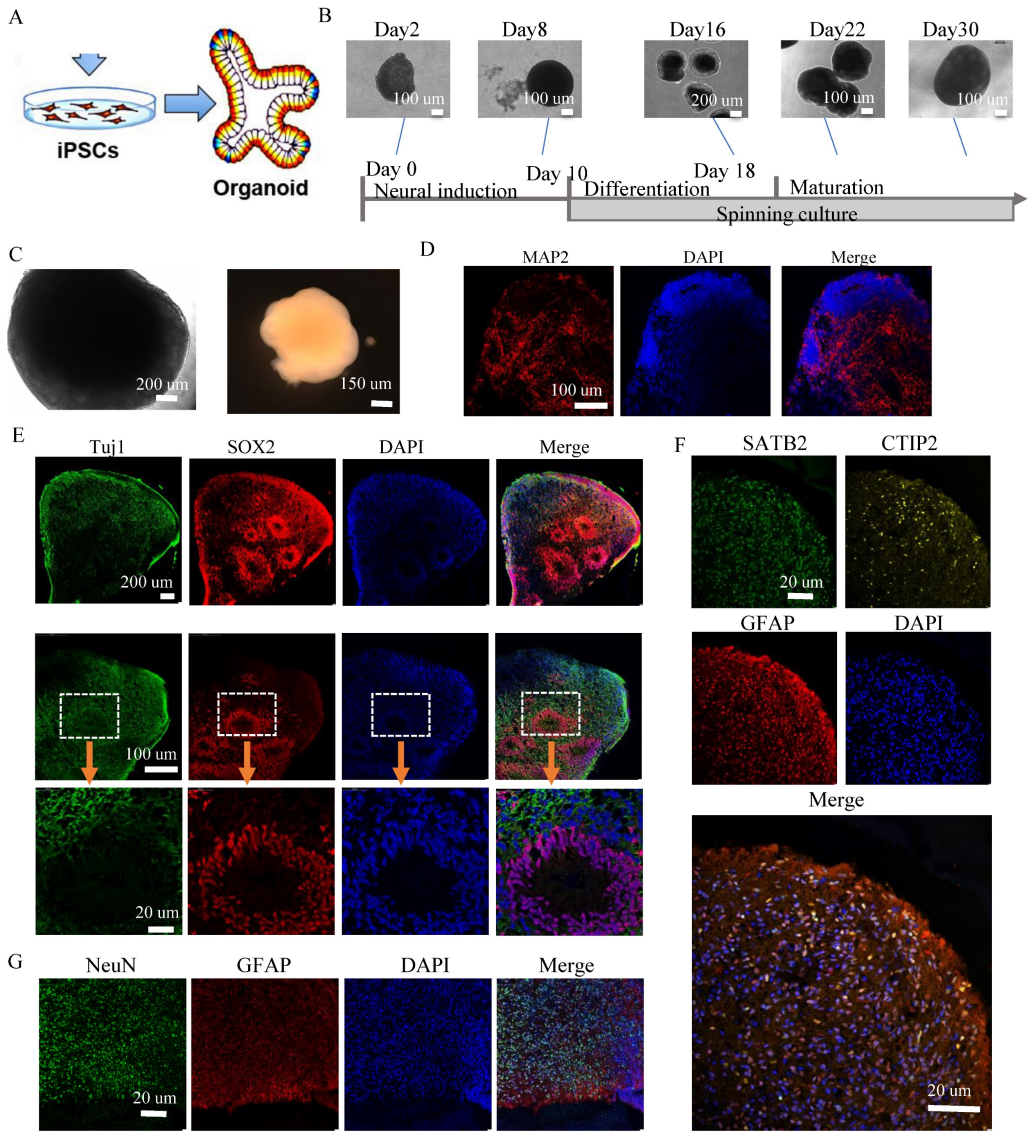
** Generation of cortical brain organoid from iPSCs.** A. Illustration of generation of cortical brain organoid. B. Generation of cortical brain organoid using iPSCs. Scale bar is 100 µm or 200 µm. C. Phase-contrast images of cortical organoids of Alpers' patient and controls at day 54. Scale bar is 200 µm (left) or 150 µm (right). D. Fluorescent staining of cortical organoid section using mature neuron marker MAP2 at day 40. Nuclei are stained with DAPI (blue). Scale bar is 20 µm. E. Fluorescent staining of cortical organoid section using neuron marker Tuj1 and neural progenitor marker SOX2 at day 40. Nuclei are stained with DAPI (blue). Scale bar is 20 µm. F. Fluorescent staining of cortical organoid section using cortical neuronal markers SATB2, CTIP2 and astrocyte marker GFAP at day 90. Nuclei are stained with DAPI (blue). Scale bar is 20 µm. G. Fluorescent staining of cortical organoid section using neuron marker NeuN and astrocyte marker GFAP at day 90. Nuclei are stained with DAPI (blue). Scale bar is 20 µm.

In our next step, we delved deeper into the mitochondrial changes in 3D cortical brain development, employing a cortical organoid model. This is a relatively complex 3D model that more accurately replicates the microenvironment and structure of the human brain (Figure 4A). Using a previously documented procedure, we transformed iPSCs into cortical organoids (Figure 4B) 29. By days 40-50, these cortical organoids had matured into large (2-3 mm in diameter) and complex heterogeneous tissues (Figure 4C). At day 40, brain organoids presented intricate morphology, including cortical folded surfaces and sulci, and expressed the mature neural marker MAP2 (Figure 4D), neonatal neural marker tubulin β III (Tuj1), ventricular zone (VZ) marker SOX2 (Figure 4E and Supplemental Figure 7). By day 90, a majority of neurons in the organoids were positive for mature neural marker NeuN in outer layers (Figure 4G and Supplemental Figure 13). Additionally, a minor fraction of cells in the organoids were GFAP positive (Figure 4F, G and Supplemental Figure 13). Furthermore, the cortical pyramidal neuronal marker CTIP2 and SATB2 were also expressed in the upper layers and middle layer of the organoids (Figure 4F and Supplemental Figure 13), demonstrating that the organoids' resemblance to cortical neurons.

**Figure 5:**
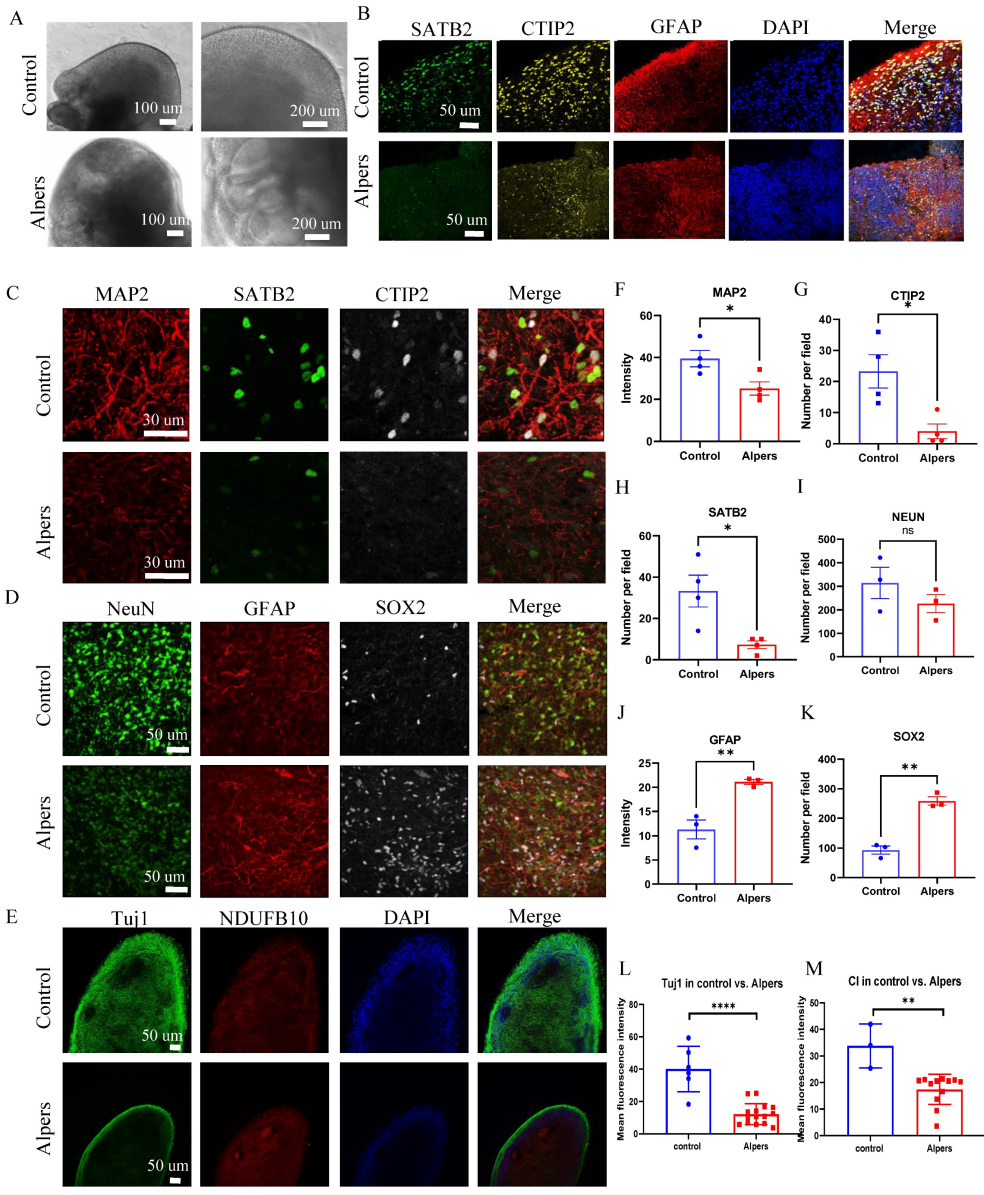
** Cortical layer deformation, neuronal loss, and astrocyte proliferation in Alpers' cortical organoid.** A. Phase-contrast images of cortical organoids of Alpers' patient and controls at day 90. Scale bar is 100 µm or 200 µm. B. Fluorescent staining of Alpers' and control cortical organoid section using cortical neuronal markers SATB2, CTIP2 and astrocytes marker GFAP at day 90. Nuclei are stained with DAPI (blue). Scale bar is 50 µm. C. Fluorescent staining of Alpers' and control cortical organoid (day 90) section using neural fiber marker MAP2 and cortical neuronal markers SATB2, CTIP2. Nuclei are stained with DAPI (blue). Scale bar is 30 µm. D. Fluorescent staining of Alpers' and control cortical organoid (day 90) section using astrocyte marker GFAP, neural marker NEUN, and neural progenitor marker SOX2. Nuclei are stained with DAPI (blue). Scale bar is 50 µm. E. Fluorescent staining of Alpers' and control cortical organoid (day 90) section using mitochondrial CI marker NDUFB10 and neural marker Tuj1. Nuclei are stained with DAPI (blue). Scale bar is 50 µm. F-M. Quantification of immunofluorescent staining of MAP2, CTIP2, SATB2, NEUN, GFAP, SOX2, Tuj1 and CI in Alpers' cortical organoids compared with controls. Nuclei are stained with DAPI (blue). Scale bar is 50 µm. The numbers of clones and replications in each experiment were listed in Table S 14.

We then generated cortical organoids from iPSCs from Alpers' patient. Immunofluorescent staining demonstrated the neural marker MAP2 expression, neural progenitor marker SOX2 and astrocyte marker GFAP (Supplementary Figure 8, 9) with clear neural rosette structures (Supplementary Figure 10, 11), indicating the succeed of generation of Alpers' patient cortical organoids. When compared to controls, Alpers' organoids exhibited irregular folding without typical cortical layers (Figure 5A and Supplemental Figure 12). This suggested abnormal cortical development in Alpers' organoids. Upon performing immunofluorescent staining of cortical organoids, we found that while normal control cortical organoids formed a typical cortical layer structure characterized by prominent cortical neurons, including SATB2- and CTIP2-positive neurons. Further quantification of cortical neurons, as well as NeuN and mature neurons in mature cortical organoids (day 90) revealed significantly lower expression of SATB2 and CTIP2 in Alpers' organoids (Figure 5B, C, G, H) and the level of all neural marker MAP2, NeuN and Tuj1 expression (Figure 5C, D, E, F, I, L and Supplementary Figure 14) was also lower in Alpers' organoids than in, confirming a loss of cortical neurons in Alpers' cortical organoids. Meanwhile, we observed an increase in SOX2-positive neural progenitors in Alpers' organoids compared with controls (Figure 5D, K). Previous studies have indicated that loss of mtDNA leads to a reactive increase in astrocytes 30. To further explore an astrocyte differentiation in Alpers' organoids, we examined the number of astrocytes in mature cortical organoids (day 90) and found that the number of GFAP-positive astrocytes in Alpers' organoids was significantly higher than in controls (Figure 5B, D, J and Supplementary Figure 14). This suggests that astrogliosis accompanying cortical neuronal loss is a characteristic of cortical brain pathology in Alpers' disease. Additionally, we used immunofluorescence to measure mitochondrial markers and discovered significantly reduced levels of the CI subunit NDUFB10, TFAM as a measurement of mtDNA copy numbers, and mitochondrial mass marker VDAC in Alpers' organoids compared to the controls (Figure 5E, M and Supplementary Figure 15).

These results affirm our ability to differentiate 3D cortical organoids from iPSCs derived from Alpers' patient, demonstrating that these organoids recapitulate disease-specific pathological and molecular features similar to those observed in the patient's brain.

### Alpers' cortical organoids exhibited significant downregulation of mitochondrial and synaptogenesis-related pathways, while there was an upregulation of pathways associated with astrocyte/glial cells and neuroinflammation

**Figure 6:**
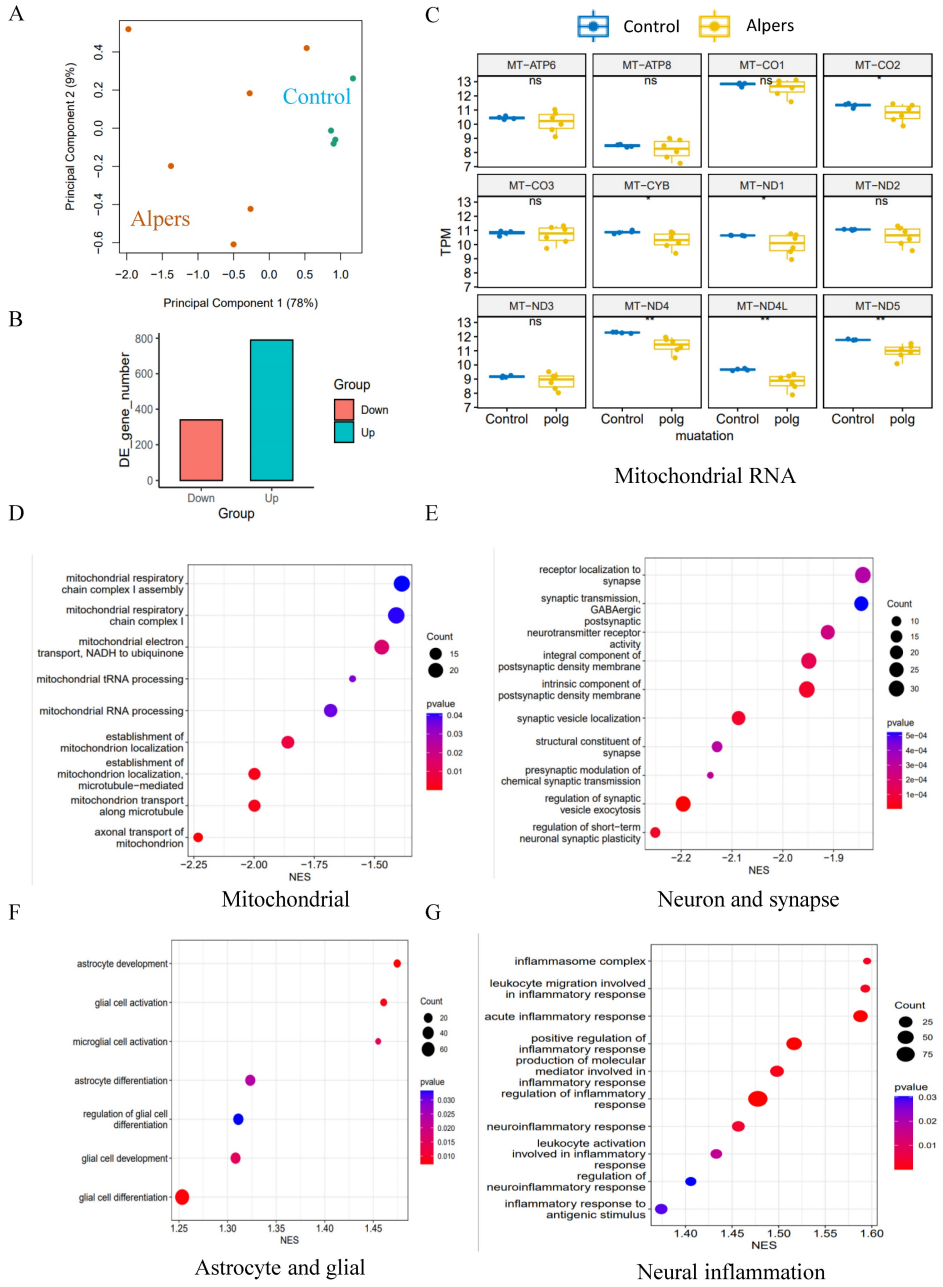
** Transcriptomic status and dysregulated pathways of Alpers' patient derived cortical organoids.** A. PCA analysis of transcriptomic expression data for Alpers' patient derived cortical organoids and controls. B. Number of upregulated and down-regulated DE genes in Alpers' cortical organoids compared with controls. C. RNA expressions of Mitochondrial encoded genes in Alpers' cortical organoids. D. Mitochondrial related pathways were downregulated in Alpers' cortical organoids. E. Synapse maturation related pathways were downregulated in Alpers' cortical organoids. F. Astrocytes and glial related pathways were upregulated in Alpers' cortical organoids. G. Neural inflammation related pathways were upregulated in Alpers' cortical organoids.

To further explore regulatory pathways in Alpers' cortical organoids, we performed RNA-seq analysis on both Alpers' and control organoids. We selected 90-day-old cortical organoids for this analysis because, as mentioned earlier, they display a relatively mature state with a layered structure of cortical neurons and glial cells (Figure 4B, G, I and Supplemental Figure 9). The PCA analysis demonstrated that Alpers' and control organoids formed distinct clusters (Figure 6A), suggesting a divergence in expression profiles between the two groups. DEGs analysis revealed 1440 differentially expressed genes, with 1010 genes being up-regulated and 430 genes down-regulated in Alpers' organoids compared to the control group (Figure 6B). Furthermore, when comparing the expression of mitochondrial transcripts in the Alpers' group to the control group, significant decreases in the expressions of *MT-CO2*,* MT-CYB*,* MT-ND1*,* MT-ND4*,* MT-ND4L*, and *MT-ND5* were noted in Alpers' organoids (Figure 6C).

Gene set enrichment analysis (GSEA) results indicated that Alpers' organoids exhibited substantial downregulation of mitochondrial and synaptogenesis-related pathways. This includes mitochondrial-related pathways including mitochondrial CI, mitochondrial electron transport, mitochondrial tRNA processing, and mitochondrial localization and trafficking (Figure 6D and Table S3), as well as axonal trafficking and synapse-related pathways including presynaptic transmission, synaptic vesicle exocytosis, and postsynaptic receptor activity (Figure 6E and Table S4). GABAergic neurons, inhibitory interneurons crucial in preventing epilepsy 31, showed marked downregulation of their synaptic transmission pathway in Alpers' cortical organoids (Figure 6E and Table S4), indicating pathological changes of GABAergic neurons in Alpers' disease. The immunostaining results for the interneuron marker GAD 65 align with this, as there was a notable reduction in GAD 65 expression in the organoids derived from Alpers' patient, compared to the controls (refer to Supplementary Figure 16). Moreover, astrocyte- and glial-related pathways were upregulated in Alpers' cortical organoids (Figure 6F and Table S5), consistent with the immunostaining findings described earlier (Figure 5D, J). Intriguingly, cortical organoids from Alpers' patient also demonstrated an up-regulation of neuroinflammatory pathways (Figure 6G and Table S6).

These findings suggest that Alpers' cortical organoids display a distinct transcriptomic profile, characterized by the downregulation of mitochondrial and synaptogenesis-related pathways and upregulation of astrocyte/glial-related and neuroinflammatory pathways.

### Long-term treatment with NR partially ameliorated the neurodegenerative alterations observed in Alpers' cortical organoids

NAD^+^ is a crucial metabolite that plays pivotal roles in cellular energy metabolism, genomic stability, and mitochondrial balance, with impacts on neurogenesis and the organization of neuronal networks 32. Prior research has highlighted that the enhancement of NAD^+^ levels through supplementation can significantly bolster mitochondrial function, positioning NAD^+^ as a promising neuroprotective agent 33. In alignment with these findings, our results illustrated a severe disruption of the NADH pathway and mitochondrial function in the iPSC-NSCs model of Alpers' syndrome compared to the control group.

**Figure 7:**
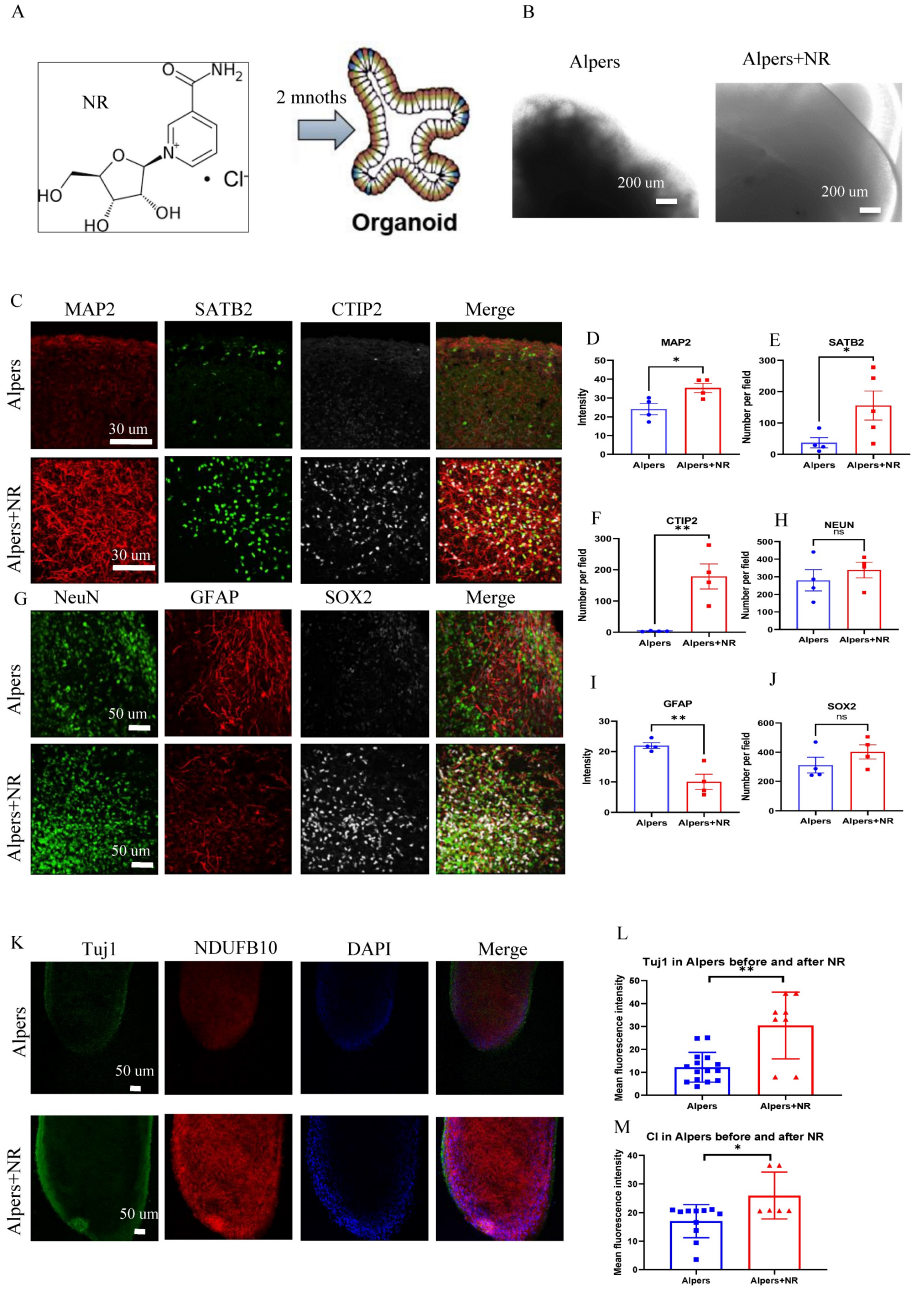
** Long time treatment of NR partially rescues changes of Alpers' cortical organoids.** A. Illustration of treatment of NR of Alpers' cortical organoids. B. Phase-contrast images of NR treatment of cortical organoids of Alpers' patient. Scale bar is 200 µm. C. Fluorescent staining of NR treated Alpers' cortical organoid section using neural fiber marker MAP2 and cortical neuronal markers SATB2, CTIP2. Nuclei are stained with DAPI (blue). Scale bar is 30 µm. D-F. Quantification of immunofluorescent staining of MAP2, SATB2 and CTIP2 in NR treated Alpers' cortical organoids compared with organoids without treatment. G. Fluorescent staining of NR treated Alpers' cortical organoid section using astrocyte marker GFAP, neural marker NeuN, and neural progenitor marker SOX2. Nuclei are stained with DAPI (blue). Scale bar is 50 µm. H-J. Quantification of immunofluorescent staining of NeuN, GFAP and SOX2 in NR treated Alpers' cortical organoids compared with organoids without treatment. K. Fluorescent staining of NR treated Alpers' cortical organoid section using complex I marker NDUFB10 and neural marker Tuj1. Nuclei are stained with DAPI (blue). Scale bar is 50 µm. L-M. Quantification of immunofluorescent staining of Tuj1 and complex I marker NDUFB10 in NR treated Alpers' cortical organoids compared with organoids without treatment. The numbers of clones and replications in each experiment were listed in Table S 14.

To test the potential of NAD^+^ supplementation in mitigating this dysfunction, we administered NR, a form of NAD^+^, to Alpers' patient derived cortical organoids that were one month old, over a two-month period (Figure 7A). We observed that the NR-treated organoids exhibited organized structures (Figure 7B) and increased expression of MAP2 and Tuj1 compared to those untreated (Figure 7C, D, K, L), alongside an elevation in the cortical neural markers SATB2 and CTIP2 (Figure 7C, E, F). Interestingly, a decrease in the number of GFAP-positive astrocytes was noticed post NR treatment when compared to untreated organoids (Figure 7G, I). Immunostaining of the mitochondrial markers showed a marked rise in NDUFB10 expression and TFAM level, but decreased VDAC expression after the treatment with NR (Figure 7K, M and Supplementary Figure 17). The expression of the interneuron marker, GAD 65, demonstrated a significant increase following treatment with NR, as evidenced by immunostaining (Supplementary Figure 18).

**Figure 8:**
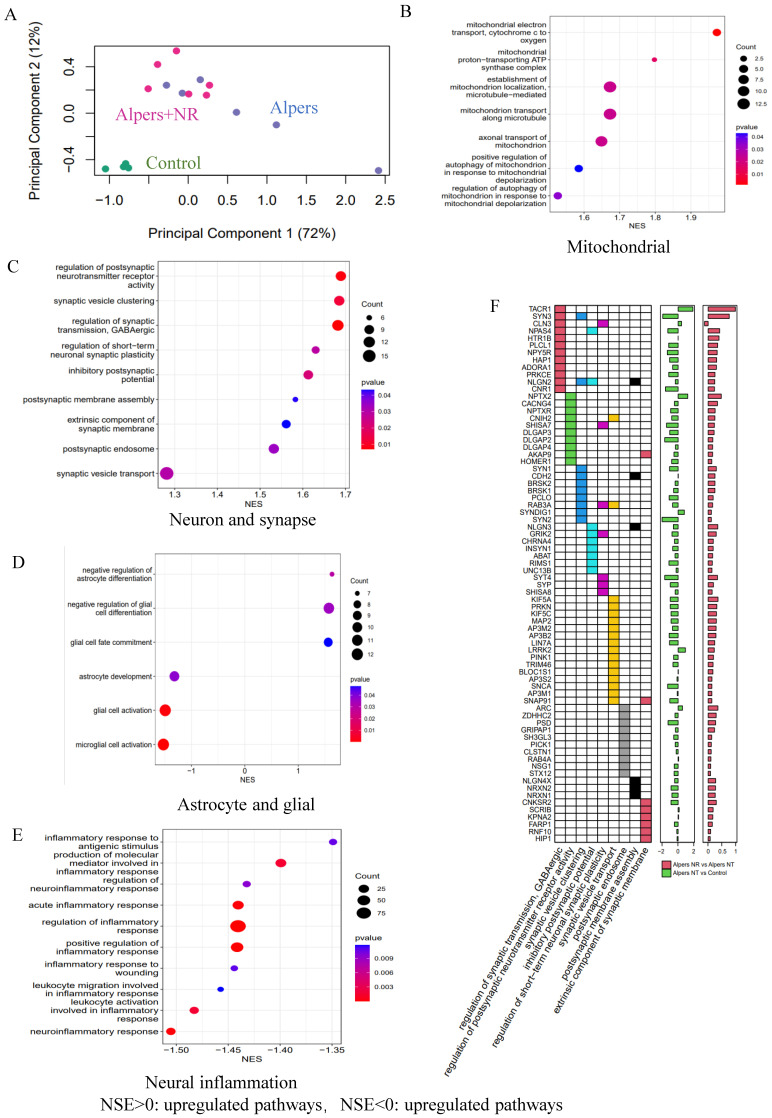
** Transcriptomic data shows the treatment effect of NR on Alpers' cortical organoids.** A. PCA analysis of transcriptomic expression data for NR, non-NR treated Alpers' cortical organoid and healthy controls. B. Mitochondrial related pathways were upregulated in NR treated Alpers' cortical organoids. C. Synapse maturation related pathways were upregulated in NR treated Alpers' cortical organoids. D. Astrocytes and glial related pathways were downregulated in NR treated Alpers' cortical organoids. E. Neural inflammation related pathways were downregulated in NR treated Alpers' cortical organoids. F. Heatmap showing the genes that were involved in significantly enriched synapse formation related pathways in NR treated Alpers' cortical organoids. X-axis on right panel represents log transformed fold change.

An RNA-seq analysis was performed to identify genetic changes before and after NR treatment. PCA demonstrated that NR treatment altered the RNA expression profile of Alpers' organoids significantly, moving it closer to that of healthy controls (Figure 8A). GSEA showed that NR treatment was able to reverse the dysregulated pathways that were identified in Alpers' patient organoids. While mtDNA RNA levels did not see a significant rise with NR treatment (Supplemental Figure 19), we noted an up-regulation in the activity of pathways related to mitochondrial function, such as mitochondrial electron transport, proton transport ATP synthase complex, mitochondrial transport along microtubules and axons, and mitophagy in response to mitochondrial depolarization (Figure 8B and Table S7).

Further, patient organoids treated with NR demonstrated an upregulation of pathways associated with synapses, including postsynaptic pathways (membrane assembly, endosomal and neurotransmitter receptor activity), inhibitory neuronal pathways (regulator of GABAergic synaptic transmission, inhibitory postsynaptic potentials), and synaptic vesicles (synaptic vesicle aggregation and trafficking) (Figure 8C, F and Table S8). In contrast, astrocyte and glial cell pathways were downregulated after NR treatment, including astrocyte development, glial cell activation, glial cell fate commitment, and pathways negatively regulating astrocyte/glial cell differentiation (Figure 8D and Table S9). Intriguingly, we also observed a downregulation of pathways related to neuroinflammation following NR treatment (Figure 8E and Table S10).

Taken together, these findings suggest that the supplementation of NAD^+^, specifically in the form of NR, could be an effective therapeutic strategy to counteract neuronal loss, glial enrichment, and mitochondrial damage observed in the cortex of Alpers' syndrome patient.

## Discussion

The ability to reprogram patient cells and differentiate iPSCs into the affected cell types has been transformative for the field 34, 35. In our research, we converted fibroblasts from an Alpers' syndrome patient with *POLG* mutations into iPSCs, and then into NSCs and 3D cortical organoids. These models successfully replicated the patient's brain pathology, showing mitochondrial DNA depletion and CI deficiency. In addition, the 3D cortical organoids manifested changes akin to those observed in the patient's cerebral cortex, including cortical neuronal loss and astrogliosis 36. This approach led to new insights into the disease mechanisms, such as increased ROS production and faulty NAD^+^ metabolism. Notably, NAD^+^ supplementation improved these conditions, offering potential avenues for future diagnostics and treatments in POLG-related diseases.

Coupled with our finding of decreased mtDNA transcript levels in Alpers' iPSCs, the reduced CI levels exhibited in the Alper's iPSCs suggests that mtDNA depletion in these Alpers' does not result in significant mitochondrial dysfunction. One possible explanation for this observation is iPSCs' higher reliance on glycolysis for iPSC energy productivity than oxidative phosphorylation 37.

Given that neuronal cells, including NSCs, rely more heavily on mitochondrial OXPHOS for their energy needs 38, iPSCs from Alpers manifested a more pronounced impairment of mitochondrial function upon differentiated into neurons. In these Alpers NSCs we observed a lower MMP, decreased ATP production, and elevated ROS generation. We also noted significant reductions in mitochondrial CI, III, IV protein levels and mtDNA transcripts. As potential precursors to cellular stress leading to apoptosis, decreased levels of mtDNA transcripts may play a key role in the development of neurological diseases. Our research thus illustrates that the impacts of mtDNA loss in Alpers' mutations start to become evident upon differentiation into NSCs. This indicates that NSCs serve as an effective model for studying diseases related to Alpers' mutations.

Our results also highlight the intricate relationship between mitochondrial dysfunction and the induction of apoptosis in Alpers' NSCs. The observed downregulation of key pro-apoptotic genes (*CASP3*, *CASP9*, and *CYCS*) alongside the upregulation of anti-apoptotic factors (*BCL2*, *BCL2L1*, *MCL1*) suggests that Alpers' NSCs may initiate adaptive mechanisms to counteract the mitochondrial impairment and the consequent elevation in ROS production. These adaptations appear to temporally mitigate the activation of apoptosis, potentially delaying cell death. Moreover, the upregulation of genes regulated by the NRF2-mediated oxidative stress response pathway (*HMOX1*, *NQO1*) further supports the notion that NSCs are actively engaging in defense mechanisms against oxidative stress. The significant downregulation of mitochondrial CI, III, IV protein levels and mtDNA transcripts could contribute to a bioenergetic crisis that exacerbates the vulnerability of Alpers' NSCs, reinforcing the need for reliance on alternative metabolic pathways such as glycolysis.

The analysis of transcriptomic data in Alpers' organoids disclosed a significant decrease in the expression of mtDNA transcripts within Alpers' organoids and the downregulation of mitochondrial-associated pathways, findings that align with those derived from NSCs. Of note, neuron- and synapse-related pathways demonstrated significant down-regulated, with GABAergic inhibition of neuron-related synaptic pathways being particularly affected. This suggests that neuronal interconnections may be compromised in Alpers' syndrome, a conclusion that aligns with previous findings regarding brain pathology in Alpers' patients 39. These results propose that cortical neuronal loss, as a secondary effect to mtDNA depletion and compromised ATP export in Alpers' patients, might contribute to central nervous system symptoms observed in these patients, including epilepsy and dementia.

The irregular morphology, atypical cortical layers, and irregular folding of Alpers' organoids underscore aberrant cortical development, potentially contributing to the clinical manifestation of Alpers' syndrome. A significant reduction of cortical neuronal marker and mature neural marker in the organoids signifying the loss of cortical neurons. Additionally, the overexpression of SOX2-positive neural progenitors and an increased number of GFAP-positive astrocytes point towards astrogliosis in the context of cortical neuronal loss, a hallmark of brain pathology in Alpers' syndrome. These findings align with the understanding that Alpers' syndrome is a neurodegenerative disorder characterized by the premature loss of neurons, particularly within the cortex, and the concurrent proliferation of astrocytes (a process known as astrogliosis) in response to neuronal damage 40. Further, the reduced expression of CI subunit NDUFB10 strengthens the notion of impaired mitochondrial function in Alpers' disease, which aligns with findings from previous studies conducted on postmortem brain tissue 39.

The differential gene expression observed through RNA-seq analysis gives a more detailed view of the altered molecular landscape within Alpers' organoids. PCA revealed a clear distinction in gene expression profiles between Alpers' and control organoids. This finding is consistent with previous research highlighting the distinct genetic signatures associated with neurodegenerative disorders 41, including Alpers' syndrome. Of particular note, the downregulation of mitochondrial transcripts implies impaired mitochondrial function in Alpers' disease, in agreement with the postulated role of mitochondrial dysfunction in this condition 39. The GSEA further uncovered dysregulated pathways in Alpers' organoids. The downregulation of mitochondrial and synaptogenesis-related pathways, including presynaptic transmission, synaptic vesicle exocytosis, and postsynaptic receptor activity, could underlie the neurological symptoms seen in Alpers' syndrome, such as seizures, developmental regression, and cognitive decline. This is especially evident with the observed downregulation of GABAergic synaptic transmission pathway, given the key role of GABAergic interneurons in epilepsy pathogenesis 42. In contrast, the upregulation of astrocyte- and glial-related pathways, coupled with the evidence of increased astrocyte differentiation, suggests reactive astrogliosis in Alpers' syndrome. Furthermore, the upregulation of neuroinflammatory pathways may reflect an innate immune response to ongoing neuronal damage and loss, an aspect that warrants further exploration. Taken together, the insights derived from these 3D cortical organoids offer a promising avenue for exploring the complex pathophysiology of Alpers' syndrome, facilitating the development of potential therapeutic interventions. Moreover, the upregulation of neuroinflammatory pathways could represent a novel target for therapeutic strategies, given the increasing evidence for the involvement of neuroinflammation in neurodegenerative disorders 43, 44.

In this study, the role of NAD^+^ supplementation, specifically the administration of NR, was investigated as a potential therapeutic strategy to alleviate the pathological changes in Alpers' syndrome. NR supplementation has previously been shown to improve mitochondrial function 45, restore NAD^+^ levels, and have a neuroprotective effect 46. These findings provided the rationale for investigating the potential benefits of NR supplementation in an Alpers' patient derived iPSC model, which revealed profound mitochondrial dysfunction. Upon administering NR to the patient derived cortical organoids, notable improvements were observed. Specifically, an increased expression of mature neuronal marker (MAP2) and cortical neural markers (SATB2 and CTIP2) was seen, indicating a recovery of neuronal populations in the NR-treated organoids. Additionally, a decrease in the number of GFAP-positive astrocytes, a marker for astrogliosis, was observed, further substantiating the neuroprotective effects of NR 45. These morphological changes corresponded with the recovery of mitochondrial function, as evidenced by the increased expression of the CI subunit NDUFB10 in NR-treated organoids. RNA-seq analysis offered further insights into the molecular underpinnings of NR's therapeutic effect. PCA analysis revealed that the entire RNA expression profile of the NR-treated Alpers' organoids shifted closer to that of the healthy controls, implying that NR treatment significantly altered gene expression. The GSEA pathway analysis corroborated these results, demonstrating upregulation of mitochondrial-related pathways and synaptic pathways, and downregulation of astrocyte/glial cell pathways and neuroinflammatory pathways in the NR-treated organoids. These findings lend credence to the potential therapeutic efficacy of NAD^+^ supplementation in Alpers' syndrome.

In discussing the limitations of this study, it's worth emphasizing the challenges posed by the inherent rarity of Alpers' syndrome. Acquiring a large sample size of patient derived iPSC lines for our experiments was a significant hurdle due to the scarcity of patients, coupled with the complexities involved in reprogramming patient fibroblasts into iPSCs. Out of the two patient iPSC lines initially available to us, one demonstrated poor growth during its differentiation into brain organoids, likely an outcome of impaired mitochondrial function. This line failed to form embryoid bodies during the early stages of organoid differentiation and was unable to withstand the cryopreservation process. Consequently, our experiments were conducted on two individual clones.

Furthermore, we understand the critique raised concerning the inclusion of additional patient lines or isogenic controls to bolster our conclusions. To this end, we attempted to use CRISPR on the patient's iPSCs to generate isogenic controls. Unfortunately, the patient's iPSCs exhibited suboptimal growth conducive to this procedure. As a solution, we decided to incorporate three different controls with multiple clones for each line, culminating in a total of five clones for the analysis. While this somewhat addressed the issue, it is nevertheless a limitation of our study, and results should be interpreted with this in mind.

Thus, although our study has yielded significant insights into the pathophysiological progression of Alpers' disease and potential therapeutic pathways, it is important to consider the constraints tied to the limited number of patient iPSC lines and the absence of isogenic controls. Future research may benefit from advancements in methodologies and techniques that allow for more efficient generation and growth of patient derived iPSCs, as well as more successful incorporation of isogenic controls. Despite these limitations, we believe our study offers a meaningful contribution to the understanding and potential treatment of Alpers' disease.

In conclusion, our research underscores the applicability of iPSC derived NSC and cortical organoid models as representative systems to simulate the pathophysiological progression of Alpers' disease. These platforms stand as robust tools for facilitating the discovery of therapeutic strategies for such debilitating conditions. Moreover, we provide evidence that NAD^+^ supplementation, specifically through NR therapy, could present a promising clinical avenue for neuroprotection not only in Alpers' disease, but also in a broader spectrum of mitochondrial disorders.

## Materials and Methods

### Ethics approval

The project was approved by the Western Norway Committee for Ethics in Health Research (REK 2012/919).

### Generation of iPSCs and NSCs

The fibroblasts from two Alpers' patient (male, 8 months/2 years old) carrying compound heterozygous *POLG* mutations A467T (c.1399G>A), P589L (c.1766C>T) were collected by punch biopsy. Three individual clones of Detroit 551 fibroblasts (ATCC CCL 110TM, female/fetus), one clone of CRL2097 fibroblasts (ATCC CRL-2097™, male/newborn) and one clone of AG05836 (CVCL_2B58, female/44 years old) were used as control individual. To generate iPSCs, skin fibroblasts were infected with Sendai virus vectors containing coding sequences of human OCT4, SOX2, KLF4, and c-MYC as described previously 28, 47. All iPSC lines were maintained under feeder-free conditions using Geltrex (Life Technologies) in E8 medium (Life Technologies) in 6-well plates (Thermo Fisher Scientific). Each line was passaged with 0.5 mM EDTA (Life Technologies) at 70-80% confluency. The E8 was changed every day, and the cells passaged every 3-4 days. All the cells were monitored for mycoplasma contamination regularly using MycoAlert™ Mycoplasma Detection Kit (Lonza). The process of generating NSCs iPSC was carried out in accordance with the methodology detailed in a previous publication 28.

### Cortical organoid generation

To generate cortical organoids from iPSCs, we used the protocol described previously 29. Briefly, feeder-free iPSCs were fed daily with E8 medium for at least 7 days before differentiation. Colonies were dissociated using Accutase (Life Technologies) in PBS (1:1) for 10 min at 37 °C and centrifuged for 3 min at 300 × g. A total of 9,000 live cells were then seeded to 96-well ultra-low attachment tissue culture plates (Thermo Fisher Scientific) in 150 μl neural induction media and kept in suspension under rotation (95 rpm) in the presence of 50 μM ROCK inhibitor (Stem cell Technologies) and kept in suspension under rotation (85 rpm) for 24 h to form embryoid body (EB). To minimize un-directed differentiation, dual SMAD inhibition and canonical WNT inhibition are adopted during this period. On day 2, half of the media was replaced by human neural induction media in the presence of 50 μM ROCK inhibitor (Y-27632) to each well. On days 4, 6, and 8, 100 μl medium was replaced with 150 μl neural induction without ROCK inhibitor. After 10 days, the organoids were transferred into 6-well ultra-low attachment tissue culture plates (Life Sciences) in neural differentiation media minus vitamin A for the next 8 days using an orbital shaker to induce cortical organoids. On day 18, the organoids were subsequently matured in neural differentiation media with vitamin A with media changes every 3-4 days. BDNF and ascorbic acid were supplemented to facilitate long-term neural maturation.

### NR treatment of cortical organoids

NR was kindly provided by Evandro Fei Fang, University of Oslo, Norway. One-month-old organoids were treated with 1 mM NR for 2 months. NR was added to the medium and replaced every three days.

### Immunofluorescence staining of iPSCs and NSCs

Mounted sections were incubated for 1 h at room temperature with and blocked using 1 X PBS supplemented with 10% normal goat serum or 5% Bovine Serum Albumin (BSA) and 0.1% Triton X-100 and then incubated with primary antibodies diluted in blocking solution overnight at 4°C. The primary antibodies, as listed in Table S11, were used for immunostaining: Alexa Fluor Dyes (Life Technologies) were used at 1:800 dilution as secondary antibodies, as listed in Table S12. Slides were mounted using ProLong^TM^ diamond antifade mounting medium with DAPI (Life Technologies) and analyzed using the Leica TCS SP8 confocal microscope (Leica Microsystems) or Dragonfly Confocal Microscope (Andor).

### Snap-freezing and embedding of cortical organoids

Each human cortical organoid was fixed in 4% PFA in PBS overnight at 4°C, dehydrated with 30% sucrose in phosphate-buffered saline (PBS). The samples were then embedded int gelatin solution (Sigma-Aldrich) and snap frozen in nitrogen. The samples were then embedded in O.C.T. compound (Thermo Fisher Scientific). Cryostat sections (15 µm) were cut and mounted onto slides (Thermo Fisher Scientific).

### Immunofluorescence staining of cortical organoids

The organoids were transferred from the culture onto a Superfrost™ adhesion slide using a 1 ml pipette with a cutting tip to ensure intact transfer without disruption. Excess medium was removed, and the slide was allowed to dry completely. The organoids were fixed with 4% (v/v) EM grade paraformaldehyde (PFA) in 1X PBS for 30 minutes at room temperature. The PFA solution was aspirated, and the slide was washed twice with 1X PBS. To prevent non-specific binding of antibodies and facilitate cell permeabilization, a blocking buffer containing 10% (v/v) normal goat serum and 0.1% (v/v) Triton X100 in 1X PBS was added to the slide and incubated at room temperature for 2 h. A circle was drawn with a Liquid Blocker pen to confine the blocking buffer within the samples. Primary antibodies were prepared in blocking buffer at the appropriate concentrations. The blocking buffer containing primary antibodies was added to completely cover the samples on the slide. The samples were then incubated in the dark for 48 h at 4°C. After incubation, the primary antibodies were aspirated, and the samples were washed with 1X PBS through repeated rinsing for 2 h. Secondary antibodies, along with Hoechst 33324 nuclear counterstain (1:5000), prepared in blocking buffer solution, were added to the samples and incubated for 48 h in the dark at 4°C. The concentrations and types of secondary antibodies used are specified in Table S12. The remaining buffer was aspirated, and the samples were washed with 1X PBS through quick repetitive rinsing. Subsequently, the samples were kept in PBS containing 0.01% (v/v) sodium azide and incubated overnight at 4°C in the dark to prevent contamination. The following day, the solution was removed, and the samples were mounted by adding 20 μl of Fluoromount-G mounting medium onto each sample and covering them with a 1.5 mm coverslip. The mounting medium was allowed to polymerize at room temperature in the dark for 24 h and then stored at -20°C until imaging. This process ensured the preparation and preservation of the organoid samples for subsequent imaging analysis. The antibodies were listed in Table S11 and S12.

### Quantification and image analysis

Image analysis and fluorescent signal quantification were performed with ImageJ software. Images were captured by identifying areas with well-defined and brightly stained DAPI nuclei. The Z-level was adjusted to capture the outermost layer where the staining efficiency appeared optimal. For each organoid, a minimum of 5 images from distinct regions were captured.

The analysis of fluorescent intensity was conducted using ImageJ software. Firstly, single-channel images were imported into ImageJ and converted to 8-bit grayscale using the "Image > Type > 8-bit" function. This step was necessary to utilize the "Threshold" feature in the subsequent process. The threshold was then set by selecting "Image > Adjust > Threshold". This feature enabled the selection of the desired fluorescence intensity threshold for pixel inclusion in the measurements.

Threshold values were determined individually for each channel based on control cortical organoids. The goal was to maximize the detection of positive signals while minimizing background fluorescence. To measure fluorescent intensity, the "Measure" function was utilized by selecting "Analyze > Measure". This process was repeated for each image to obtain fluorescent values for analysis.

To streamline and minimize the potential for human error, a macro was created within the ImageJ program. This macro allowed the automatic execution of the analysis on an image with a simple button press, eliminating the need for manual processing of each image. The "Integrated density" (IntDen) value obtained from the "Measure" function was used as the fluorescent intensity measurement.

### DNA sequencing for *POLG* mutations

Forward and backward oligonucleotide primers were used to amplify the 7 exons and 10 exons of the *POLG* gene. Automated nucleotide sequencing was performed using the Applied Biosystems™ BigDye^®^Terminator v3.1 Cycle Sequencing Kit (Life Technologies, cat. no. 4337454) and analyzed on an ABI3730 Genetic Analyzer with sequencing analyzer software Chromas^Pro^ (Technelysium Pty Ltd, Australia). DNA Chromatogram was aligned with the best matching human sequences in NCBI Trace.

### Transmission electron microscopy (TEM)

The process began by fixing cells using 4% glutaraldehyde, followed by post fixation with 1% OsO4 in a cacodylate buffer (0.1 mol/L) containing 0.1% CaCl2, maintaining the temperature at 4°C for two hours. After post fixation, the samples were stained using 1% uranyl acetate filtered through Millipore, and subsequently, a gradual dehydration was carried out using ethanol in increasing concentrations. The next steps involved infiltrating the dehydrated samples and embedding them in epoxy resin. Ultrathin sections of these embedded samples were prepared and stained again with uranyl acetate and lead citrate for enhanced contrast. Finally, these samples were examined under a transmission electron microscope (JEM-1230 JEOL).

### ROS measurement

ROS production was measured by flow cytometry. Mitochondrial mass-related intracellular ROS was detected using 30 μM DCFDA (Abcam) and 150 nM MTDR (Life Technologies). Mitochondrial ROS production in relation to mitochondrial mass was detected using 10 μM MitoSOX Red Mitochondrial Superoxide Indicator (Life Technologies) and 150 MTG (Life Technologies). double staining. After staining, stained cells were detached using TrypLE^TM^ Express enzyme (Life Technologies) and neutralized with a medium containing 10% FBS, and then analyzed on a FACS BD Accuri^TM^ C6 flow cytometer (BD Biosciences, San Jose, CA, USA). At least 40,000 events were recorded per sample, and doublets or dead cells were excluded. The results were analyzed using Accuri^TM^ C6 software.

### mtDNA copy number measurement

mtDNA copy number was quantified using flow cytometry and qPCR. For flow cytometry, cells were stained with TFAM and TOMM20. Cells were detached with TrypLE^TM^ Express enzyme and fixed with 1.6% (v/v) PFA (VWR) for 10 min at room temperature. Cells were permeabilized with ice-cold 90% methanol for 20 min at - 20°C after washing with PBS, followed by blocking buffer containing PBS, 0.3M glycine, 5% goat serum, and 1% BSA. closed. Cells were incubated with anti-TFAM antibody conjugated to Alexa Fluor^®^ 488 (Abcam) at 1:400 dilution and anti-TOMM20 antibody conjugated to Alexa Fluor^®^ 488 (Santa Cruz Biotechnology) at a 1:400 dilution. Cells were then analyzed on a BD Accuri^TM^ C6 flow cytometer. At least 40,000 events were recorded per sample, and doublets or dead cells were excluded. The data analysis was performed using Accuri^TM^ C6 software.

For qPCR, DNA was extracted using the QIAGEN DNeasy Blood and Tissue Kit (QIAGEN) according to the manufacturer's protocol and ND1/APP was measured as previously published method for relative quantification of mtDNA copy number 7.

### Mitochondrial respiratory chain complex measurement

Protein levels of mitochondrial CI, CII, and CIV were accessed using flow cytometry. Cells were detached with TrypLE^TM^ enzyme and were fixed with 1.6% (v/v) PFA (VWR) for 10 minutes at RT, before permeabilized using ice-cold 90% methanol at -20°C for 20 min. The cells were blocked using the blocking buffer mentioned above. Cells were stained with primary antibodies at dilutions 1:1000. The primary antibodies include anti-NDUFB10 (Abcam), anti-SDHA (Abcam) and anti-COX IV (Abcam). The secondary antibodies Alexa Fluor^®^ goat Anti-rabbit 488 or Anti-mouse 488 (Thermo Fisher Scientific) were subsequently incubated at dilutions 1:400. The cells were analyzed on BD Accuri^TM^ C6 flow cytometer and data analysis was performed using Accuri^TM^ C6 software. At least 40,000 events were recorded for each sample, doublets or dead cells were excluded. The antibodies were listed in Table S11 and S12.

### MMP measurement

MMPs were measured relative to mitochondrial volume using flow cytometry. Cells were double stained with 100 nM TMRE (Abcam) and 150 nM MTG (Life Technologies) for 45 min at 37 °C. Cells treated with 100 μM Carbonyl cyanide-p-trifluoromethoxyphenylhydrazone (FCCP) (Abcam) were used as a negative control. After washing with PBS, cells were detached with TrypLE^TM^ enzyme and neutralized with medium supplemented with 10% FBS. Cells were then analyzed on a FACS BD Accuri^TM^ C6 flow cytometer. Data analysis was performed using Accuri™ C6 software. At least 40,000 events were recorded per sample, and doublets or dead cells were excluded.

### ATP production

Intracellular ATP production was detected using the Luminescent ATP Detection Assay Kit (Abcam). Cells were grown to 90% confluency in 96-well plates (Life Sciences). ATP measurements were performed according to the manufacturer's protocol. After cells were lysed, luciferase and luciferin were added, and the emitted light corresponding to the amount of ATP was measured in a Victor^®^ XLight Multimode Plate Reader (PerkinElmer). For each sample, measure 3-6 replicates. To normalize cell numbers for ATP production, cells grown in the same 96-well plate were stained using the Janus Green Cell Normalization Staining Kit (Abcam). OD values at 595 nm were measured by a Labsystems Multiskan Bichromatic plate reader (Titertek Instruments, USA).

### L-lactate measurement

L-lactate production was analyzed by a colorimetric L-lactate assay kit (Abcam) according to the manufacturer's instructions. Determine the endpoint lactate concentration in a 96-well plate by measuring the initial rate (2 min) of lactate dehydrogenase equilibration between NAD^+^ and NADH. Immediately after the extracellular flux assay, plates were measured at OD 450 nm in a microplate reader (VICTOR™ XLight, PerkinElmer).

### RNA-seq analysis

Total RNA was isolated from NSCs and iPSCs using RNeasy Mini Kit (QIAGEN). RNA quality and concentration were checked using Bioanalyzer and Qubit^TM^. RNA-seq libraries were established by the PolyA enrichment method. The libraries were sequenced at a depth of over 22 million raw reads, ensuring comprehensive coverage of the transcriptome and sufficient depth for accurate and reliable analysis of gene expression. For iPSCs and NSCs, RNA-seq analysis was performed by HudsonAlpha. For brain organoid, the RNA sequencing analysis was performed by BGI. FASTQ files were trimmed using Trimmomatic version 0.39 to remove potential Illumina adapters and low quality bases with the following parameters: ILLUMINACLIP: truseq.fa:2:30:10 LEADING:3 TRAILING:3 SLIDINGWINDOW:4:15 48. FASTQ files were assessed using fastQC version 0.11.8 prior and following trimming 49. We used Salmon version 1.0.0 to quantify the abundance at the transcript level with the fragment-level GC bias correction option (*gcBias*flag) and the appropriate option for the library type (*l*flag set to A) against the GENCODE release 32 of the human transcriptome (GRCh38.p13) 50. Transcript-level quantification was imported into R and collapsed onto gene-level quantification using the tximport R package version 1.8.0 according to the gene definitions provided by the same GENCODE release 51. The genes in non-canonical chromosomes and scaffolds were filtered out. Genes were filtered out if their level of expression was below 10 reads in more than 75% of the samples based on CPM (count per million). Transcripts counts were aggregated into gene counts. Sample perason correlations were calculated with the information of library size, cell type and mutation, and sample outliers were excluded. Complete linkage hierarchical clustering method and PCA were conducted using Stats package. For PCA analysis, the first two compoments were visualized and no rotation method was specified. Differential expression analysis was conducted using DEseq2 52. Multiple comparisons were adjusted by using the false discovery rate method. Adjusted P value (q value) <0.05 was considered as statistical significance. For categorization, we considered RNAs with a log2 fold change (log2FC) greater than 0 as upregulated, and those with a log2FC less than 0 as downregulated. We opted not to set a more stringent log2FC threshold for significance. KEGG pathway and GO enrichment analysis were conducted using Clusterprofiler 53. To ensure the robustness and reliability of our results, we included three replicates for each clone in the iPSC and NSC samples. Furthermore, for the organoid samples, we included three replicates for the patient lines and four replicates for the control line. In each replicate, we analyzed 4-6 organoids for RNA sequencing, allowing us to capture the inherent biological variability within the samples.

### Statistical analysis

Data were presented as mean ± standard deviation (SD) for the number of samples (n ≥ 3). Distributions were tested for normality using the Shapiro-Wilk test and graphical method using Q-Q plot. Outliers were detected using the ROUT method. A two-sided Student's t-test was applied for normally distributed variables, which had been tested homogenous for varience across groups, while Mann-Whitney U-test was used to assess statistical significance for variables with non-normal distribution. Data were analyzed and figures were produced with GraphPad Prism 8.0.2 software GraphPad Software, Inc). The error bar of barplots refers mean/median with 95% confidence intervals. P ≤ 0.05 was considered significant (* *P*<0.05, ** *P*<0.01, *** *P*<0.001).

### Data Availability

The datasets generated and analyzed during the study are included with the Supplemental Information. The RNA-seq analysis read count data can be accessed in NCBI Gene Expression Omnibus (GEO) data deposit system with an accession number GSE207007. All other data are available from the corresponding author upon request.

## Supplementary Material

Supplementary figures and tables.
