# Supplementary material for: The NAD+ Precursor Nicotinamide Riboside Rescues Mitochondrial Defects and Neuronal Loss in iPSC derived Cortical Organoid of Alpers' Disease
Source: Int J Biol Sci. 2024 Jan 25;20(4):1194–217. doi: 10.7150/ijbs.91624 (PMC10878163; doi:10.7150/ijbs.91624)
Supplement: Supplementary file 1 — Supplementary figures and tables. [file ijbsv20p1194s1.pdf]

Table S1: The KEGG pathways of the down- and up- regulated DEGs in iPSCs from patient organoids versus controls.

ID	pathway	pvalue	geneID
iPSC downregulated pathways			
M00142	NADH:ubiquinone oxidoreductase, mitochondria	0.000729	ND1/ND4/ND5/ND4L
M00140	C1-unit interconversion, prokaryotes	0.00512	SHMT2/SHMT1
M00346	Formaldehyde assimilation, serine pathway	0.015401	ENO3/SHMT2/SHMT1
M00158	F-type ATPase, eukaryotes	0.028616	ATP5PO/ATP5PB/ATP5MC3/ATP8
iPSC upregulated pathways			
M00089	Triacylglycerol biosynthesis	0.001815	AGPAT1/LPIN2/AGPAT4/GPAT3/DGAT2/LCLAT1
M00345	Formaldehyde assimilation, ribulose monophosphate pathway	0.01069	PFKM/PFKP
M00001	Glycolysis (Embden-Meyerhof pathway), glucose => pyruvate	0.017689	PGAM2/GCK/PFKM/PFKP/PKLR
M00056	O-glycan biosynthesis, mucin type core	0.024055	GALNT2/GALNT14/GCNT4/C1GALT1C1L/GALNT18

Table S2: The KEGG pathways of the down- and up- regulated DEGs in NSCs from patient organoids versus controls.

ID	pathway	pvalue	geneID
NSC downregulated pathways			
M00095	C5 isoprenoid biosynthesis, mevalonate pathway	9.35E-06	HMGCS1/IDI1/MVK/ACAT2/MVD/HMGCR/PMVK/ACAT1
M00101	Cholesterol biosynthesis, squalene 2,3-epoxide => cholesterol	9.35E-06	SC5D/HSD17B7/NSDHL/LSS/EBP/DHCR24/CYP51A1/TM7SF2
M00849	C5 isoprenoid biosynthesis, mevalonate pathway, archaea	0.000266	HMGCS1/IDI1/MVK/ACAT2/HMGCR/ACAT1
M00102	Ergocalciferol biosynthesis, squalene 2,3-epoxide => ergosterol/ergocalciferol	0.000438	SC5D/NSDHL/LSS/CYP51A1/TM7SF2
M00917	Phytosterol biosynthesis, squalene 2,3-epoxide => campesterol/sitosterol	0.000438	SC5D/EBP/DHCR24/CYP51A1/TM7SF2
M00142	NADH:ubiquinone oxidoreductase, mitochondria	0.013183	ND3/ND1/ND2/ND4
M00374	Dicarboxylate-hydroxybutyrate cycle	0.023823	ACAT2/ACAT1
M00555	Betaine biosynthesis, choline => betaine	0.023823	ALDH7A1/CHDH
NSC upregulated pathways			
M00001	Glycolysis (Embden-Meyerhof pathway), glucose => pyruvate	7.83E-05	ENO2/ADPGK/GPI/HKDC1/GCK/TPI1/GAPDH/ALDOA/ALDOC/PGK1/PGAM1/HK2
M00892	UDP-N-acetyl-D-glucosamine biosynthesis, eukaryotes, glucose => UDP-GlcNAc	0.000467	PGM3/GPI/HKDC1/GFPT1/GCK/UAP1/HK2
M00072	N-glycosylation by oligosaccharyltransferase	0.000954	TUSC3/RPN2/STT3A/DAD1/RPN1
M00003	Gluconeogenesis, oxaloacetate => fructose-6P	0.001854	ENO2/TPI1/GAPDH/PCK2/ALDOA/ALDOC/PGK1/PGAM1
M00549	Nucleotide sugar biosynthesis, glucose => UDP-glucose	0.00225	PGM1/HKDC1/GCK/UGP2/HK2
M00019	Valine/isoleucine biosynthesis, pyruvate => valine / 2-oxobutanoate => isoleucine	0.02065	BCAT1/BCAT2
M00119	Pantothenate biosynthesis, valine/L-aspartate => pantothenate	0.02065	BCAT1/BCAT2
M00057	Glycosaminoglycan biosynthesis, linkage tetrasaccharide	0.023492	B4GALT7/B3GALT6/B3GAT3
M00079	Keratan sulfate degradation	0.023492	GLB1/GNS/HEXB
M00002	Glycolysis, core module involving three-carbon compounds	0.028201	ENO2/TPI1/GAPDH/PGK1/PGAM1
M00078	Heparan sulfate degradation	0.028979	NAGLU/GNS/IDS/IDUA
M00058	Glycosaminoglycan biosynthesis, chondroitin sulfate backbone	0.04208	CHPF/CSGALNACT2/CHPF2
M00073	N-glycan precursor trimming	0.04208	GANAB/MAN1B1/MOGS
M00141	C1-unit interconversion, eukaryotes	0.04208	MTHFD2/MTHFD1L/SHMT2
M00854	Glycogen biosynthesis, glucose-1P => glycogen/starch	0.04208	GYS1/GBE1/UGP2

Table S3: The mitochondrial related pathways of upregulated DEGs in cortical organoids from patient organoids versus controls.

ID	pathway	pvalue	core_enrichment
GO:0019896	axonal transport of mitochondrion	0.000186	TRAK1/AGBL4/FEZ1/SPAST/OPA1/ACTR10/AGTPBP1/UCHL1/MAPT/HAP1/SYBU/NEFL
GO:0034643	establishment of mitochondrion localization, microtubule-mediated	0.002215	MAP1S/TRAK1/RHOT1/AGBL4/FEZ1/SPAST/OPA1/ACTR10/AGTPBP1/MAP1B/UCHL1/MAPT/HAP1/SYBU/NEFL
GO:0047497	mitochondrion transport along microtubule	0.002215	MAP1S/TRAK1/RHOT1/AGBL4/FEZ1/SPAST/OPA1/ACTR10/AGTPBP1/MAP1B/UCHL1/MAPT/HAP1/SYBU/NEFL
GO:0051654	establishment of mitochondrion localization	0.007642	MAP1S/TRAK1/RHOT1/AGBL4/FEZ1/SPAST/OPA1/ACTR10/MARK1/AGTPBP1/MAP1B/UCHL1/MAPT/HAP1/SYBU/NEFL
GO:0006120	mitochondrial electron transport, NADH to ubiquinone	0.015314	NDUFV1/NDUFB6/NDUFAB1/NDUFS5/NDUFS7/NDUFB2/NDUFS1/NDUFAF1/ND3/NDUFA6/NDUFC2-KCTD14/ND2/NDUFA5/ND1/PINK1/NDUFA9/ND5/ND4L/ND4/SNCA
GO:0090646	mitochondrial tRNA processing	0.034206	TRMT61B/TRIT1/TRMT5/MTO1/RPUSD4/HSD17B10/PRORP/TRNT1/PUS1/ELAC2/CDK5RAP1
GO:0000963	mitochondrial RNA processing	0.036468	FASTKD2/FASTKD3/TRMT61B/TRIT1/SUPV3L1/TBRG4/TRMT5/MTO1/RPUSD4/HSD17B10/PRORP/TRNT1/PUS1/FASTKD5/ELAC2/FASTKD1/CDK5RAP1
GO:0005747	mitochondrial respiratory chain complex I	0.040214	NDUFA10/NDUFB3/NDUFA8/NDUFA4/NDUFV1/NDUFB6/NDUFAB1/NDUFS5/NDUFA12/NDUFS7/NDUFB2/NDUFS1/NDUFAF1/ND3/FOXRED1/NDUFA6/NDUFC2-KCTD14/ND2/NDUFA5/ND1/NDUFA9/ND5/ND4L/ND4
GO:0032981	mitochondrial respiratory chain complex I assembly	0.040874	NDUFAF6/TMEM126B/NDUFB6/NDUFAB1/NDUFS5/NDUFA12/NDUFS7/NDUFB2/NDUFS1/NDUFAF1/FOXRED1/NDUFAF2/NDUFA6/TIMM21/BCS1L/ND2/NDUFA5/ND1/NDUFA9/NDUFAF4/NDUFAF5/ND5/ND4

Table S4: The axon and synaptic related pathways of the downregulated DEGs in cortical organoids from patient organoids versus controls.

ID	pathway	pvalue	core_enrichment
GO:0019896	axonal transport of mitochondrion	0.000186	TRAK1/AGBL4/FEZ1/SPAST/OPA1/ACTR10/AGTPBP1/UCHL1/MAPT/HAP1/SYBU/NEFL
GO:0034643	establishment of mitochondrion localization, microtubule-mediated	0.002215	MAP1S/TRAK1/RHOT1/AGBL4/FEZ1/SPAST/OPA1/ACTR10/AGTPBP1/MAP1B/UCHL1/MAPT/HAP1/SYBU/NEFL
GO:0047497	mitochondrion transport along microtubule	0.002215	MAP1S/TRAK1/RHOT1/AGBL4/FEZ1/SPAST/OPA1/ACTR10/AGTPBP1/MAP1B/UCHL1/MAPT/HAP1/SYBU/NEFL
GO:0051654	establishment of mitochondrion localization	0.007642	MAP1S/TRAK1/RHOT1/AGBL4/FEZ1/SPAST/OPA1/ACTR10/MARK1/AGTPBP1/MAP1B/UCHL1/MAPT/HAP1/SYBU/NEFL
GO:0006120	mitochondrial electron transport, NADH to ubiquinone	0.015314	NDUFV1/NDUFB6/NDUFAB1/NDUFS5/NDUFS7/NDUFB2/NDUFS1/NDUFAF1/ND3/NDUFA6/NDUFC2-KCTD14/ND2/NDUFA5/ND1/PINK1/NDUFA9/ND5/ND4L/ND4/SNCA
GO:0090646	mitochondrial tRNA processing	0.034206	TRMT61B/TRIT1/TRMT5/MTO1/RPUSD4/HSD17B10/PRORP/TRNT1/PUS1/ELAC2/CDK5RAP1
GO:0000963	mitochondrial RNA processing	0.036468	FASTKD2/FASTKD3/TRMT61B/TRIT1/SUPV3L1/TBRG4/TRMT5/MTO1/RPUSD4/HSD17B10/PRORP/TRNT1/PUS1/FASTKD5/ELAC2/FASTKD1/CDK5RAP1
GO:0005747	mitochondrial respiratory chain complex I	0.040214	NDUFA10/NDUFB3/NDUFA8/NDUFA4/NDUFV1/NDUFB6/NDUFAB1/NDUFS5/NDUFA12/NDUFS7/NDUFB2/NDUFS1/NDUFAF1/ND3/FOXRED1/NDUFA6/NDUFC2-KCTD14/ND2/NDUFA5/ND1/NDUFA9/ND5/ND4L/ND4
GO:0032981	mitochondrial respiratory chain complex I assembly	0.040874	NDUFAF6/TMEM126B/NDUFB6/NDUFAB1/NDUFS5/NDUFA12/NDUFS7/NDUFB2/NDUFS1/NDUFAF1/FOXRED1/NDUFAF2/NDUFA6/TIMM21/BCS1L/ND2/NDUFA5/ND1/NDUFA9/NDUFAF4/NDUFAF5/ND5/ND4

Table S5: The astrocyte related pathways of the upregulated DEGs in cortical organoids from patient organoids versus controls.

ID	pathway	pvalue	core_enrichment
GO:0010001	glial cell differentiation	0.007548	S100A9/S100A8/C1QA/DLX2/DLX1/LAMC3/MYRF/NKX6-2/MAL/TLR2/ERBB3/TNFRSF1B/NRROS/GSX2/ITGB4/NKX2-2/PAX2/NKX6-1/MXRA8/LEF1/MDK/GFAP/IL6ST/C5AR1/ROR2/TGFB1/TP73/CXCR4/SPINT1/NCMAP/SH3TC2/LAMB2/CDK6/CDKN2C/TMEM98/GRN/SOX13/CDK1/SMO/DAAM2/ERBB2/ROR1/EGR2/CLCF1/PARD3/LYN/GCM1/SOX10/ADGRG6/HDAC1/HES1/TLR4/VIM/RELN/SOX8/ASPA/CNTN2/ASCL1/PAX6/LIF/NOTCH1/FGF5/SOX6/BNIP3/BMP2/PLEC/OLIG1/PRDM8/NEUROD4/ID4/SERPINE2/METRN/SOX1/SHH/STAT3/HDAC11
GO:0061900	glial cell activation	0.008097	C1QA/CCL3/ITGB2/PTPRC/TLR2/ITGAM/CTSC/C5AR1/AIF1/TLR3/GRN/SMO/TLR6
GO:0001774	microglial cell activation	0.01446	C1QA/CCL3/ITGB2/PTPRC/TLR2/ITGAM/CTSC/C5AR1/AIF1/TLR3/GRN/TLR6
GO:0021782	glial cell development	0.014601	S100A9/S100A8/C1QA/LAMC3/MYRF/NKX6-2/MAL/TLR2/NRROS/ITGB4/NKX2-2/MXRA8/MDK/GFAP/C5AR1/ROR2/NCMAP/SH3TC2/LAMB2/CDK6/GRN/SMO/ROR1/PARD3/LYN/SOX10/ADGRG6/TLR4/VIM
GO:0048708	astrocyte differentiation	0.023119	S100A9/S100A8/C1QA/LAMC3/NKX2-2/GFAP/IL6ST/C5AR1/ROR2/LAMB2/CDK6/GRN/SMO/ROR1/CLCF1/GCM1/HES1/TLR4/VIM/SOX8/CNTN2/PAX6/LIF/NOTCH1/BMP2/ID4/SERPINE2/SHH/STAT3
GO:0045685	regulation of glial cell differentiation	0.033166	DLX2/DLX1/NKX6-2/TLR2/TNFRSF1B/GSX2/NKX2-2/NKX6-1/MDK/IL6ST/TGFB1/TP73/CXCR4/SPINT1/TMEM98/CDK1/DAAM2/EGR2/CLCF1/HDAC1/HES1/ASPA/CNTN2/LIF/NOTCH1/BMP2/ID4/SERPINE2/SOX1/SHH

Table S6: The neuroinflammation related pathways of the downregulated DEGs in cortical organoids from patient organoids versus controls.

ID	pathway	pvalue	core_enrichment
GO:0050727	regulation of inflammatory response	1.22E-09	KRT1/S100A9/PLA2G2A/S100A8/APOA1/CD28/CASP4/CCL3/PLA2G7/CREB3L3/ALOX5AP/GATA3/PTGER4/NR1H4/ACP5/VAMP8/MMP9/PTPRC/HCK/IL1R1/BIRC3/LGALS1/CASP1/SYK/TLR2/FOXF1/SLAMF8/TNFRSF1B/AHSG/C3/ANXA1/PYCARD/LRRC19/ETS1/ISL1/TNFRSF11A/CCN4/NCF1/ALOX5/ADAMTS12/MGST2/GPER1/STING1/GPSM3/CD200R1/SOCS3/DUOXA2/NFKBIZ/MDK/IFI35/IL6ST/CTSC/PTGES/AGTR1/NMI/TEK/HGF/PPARG/TNFAIP3/OSMR/NUPR1/TLR3/ZFP36/PLCG2/SCGB1A1/HLA-E/APOE/PBK/CEBPA/KLF4/NOD2/AGT/LACC1/DUOXA1/TNFRSF1A/AOAH/WNT5A/GRN/METRNL/NLRX1/MYD88/TLR7/SMAD3/MAPK13/LYN/SERPINF1/PTGS2/ADORA2B/PIK3CG/PPARA/TREX1/CEBPB
GO:0002526	acute inflammatory response	9.16E-06	SERPINA1/S100A8/APOA2/CD163/CREB3L3/ALOX5AP/SERPINA3/GATA3/FN1/A2M/AHSG/C3/TNFRSF11A/IL6R/PLSCR1/IL6ST/PTGES/ASS1/B4GALT1/F3/OSMR/NUPR1/KL/HLA-E/TACR1/TREM1/ANO6/SERPINF2/PTGS2/PIK3CG/HP/CEBPB/TFRC
GO:0050729	positive regulation of inflammatory response	2.84E-05	S100A9/PLA2G2A/S100A8/CD28/CCL3/PLA2G7/CREB3L3/ALOX5AP/PTGER4/VAMP8/LGALS1/CASP1/TLR2/C3/PYCARD/ETS1/TNFRSF11A/CCN4/MGST2/GPSM3/NFKBIZ/MDK/IFI35/IL6ST/CTSC/AGTR1/NMI/OSMR/NUPR1/TLR3/PLCG2/HLA-E/CEBPA/AGT/TNFRSF1A/WNT5A/GRN/TLR7/MAPK13/PTGS2/ADORA2B/PIK3CG/CEBPB
GO:0002532	production of molecular mediator involved in inflammatory response	0.001498	H19/ALOX5AP/VAMP8/IL4R/SYK/SLAMF8/PYCARD/NCF1/ALOX5/CD36/GPSM3/CLEC7A/NOD2/SNAP23/TICAM1/ZC3H12A/GRN/APOD/ADCY7/MYD88/TLR6/LYN/PPARA/TLR4/IL17RA/NOS2
GO:0061702	inflammasome complex	0.002189	CASP4/CASP1/PYCARD/GSDMD
GO:0002523	leukocyte migration involved in inflammatory response	0.002256	S100A9/S100A8/TRIM55/SLAMF8/ALOX5/MDK
GO:0150076	neuroinflammatory response	0.003557	C1QA/CCL3/ITGB2/MMP9/PTPRC/TLR2/TNFRSF1B/ITGAM/CD200R1/CTSC/C5AR1/NUPR1/AIF1/TLR3/PLCG2/GRN/SMO/ITGB1/TLR6/PTGS2
GO:0002269	leukocyte activation involved in inflammatory response	0.016619	C1QA/CCL3/ITGB2/PTPRC/TLR2/ITGAM/CTSC/C5AR1/AIF1/TLR3/GRN/MYD88/TLR6
GO:0002437	inflammatory response to antigenic stimulus	0.028382	CD28/GATA3/HCK/SYK/A2M/CD68/C3/IL5RA/HLA-E/NOD2/HMGB2/LYN/TREX1/NPY/RHBDF2/NOTCH1/NOTCH2/GPX1
GO:0150077	regulation of neuroinflammatory response	0.030303	CCL3/MMP9/PTPRC/TNFRSF1B/CD200R1/CTSC/NUPR1/PLCG2/GRN/PTGS2
GO:0002544	chronic inflammatory response	0.032217	S100A9/S100A8/THBS1/TNFAIP3
GO:0002675	positive regulation of acute inflammatory response	0.043678	CREB3L3/ALOX5AP/C3/TNFRSF11A/IL6ST/OSMR/HLA-E/PTGS2/PIK3CG

Table S7: The mitochondrial related pathways of the upregulated DEGs in cortical organoids from patient organoids with NR treatment versus non-treated patient organoids.

ID	pathway	pvalue	core_enrichment
GO:0006123	mitochondrial electron transport, cytochrome c to oxygen	0.001645	MTCO2P12/COX2/COX1/COX3
GO:0005753	mitochondrial proton-transporting ATP synthase complex	0.014619	ATP8/ATP6
GO:0019896	axonal transport of mitochondrion	0.022516	UCHL1/SYBU/HAP1/MAPT/FEZ1/NEFL/SPAST/HSBP1/TRAK1/AGTPBP1/MGARP
GO:0034643	establishment of mitochondrion localization, microtubule-mediated	0.022935	UCHL1/SYBU/HAP1/MAPT/FEZ1/NEFL/SPAST/MAP1B/HSBP1/TRAK1/AGTPBP1/WASF1/MGARP
GO:0047497	mitochondrion transport along microtubule	0.022935	UCHL1/SYBU/HAP1/MAPT/FEZ1/NEFL/SPAST/MAP1B/HSBP1/TRAK1/AGTPBP1/WASF1/MGARP
GO:1904923	regulation of autophagy of mitochondrion in response to mitochondrial depolarization	0.034473	HK2/MFN2/PRKN/PINK1
GO:1904925	positive regulation of autophagy of mitochondrion in response to mitochondrial depolarization	0.042801	HK2/MFN2/PRKN/PINK1

Table S8: The axon and synaptic related pathways of the downregulated DEGs in cortical organoids from patient organoids with NR treatment versus non-treated patient organoids.

ID	pathway	pvalue	core_enrichment
GO:0032228	regulation of synaptic transmission, GABAergic	0.007103	TACR1/SYN3/CLN3/NPAS4/HTR1B/PLCL1/NPY5R/HAP1/ADORA1/PRKCE/NLGN2/CNR1
GO:0098962	regulation of postsynaptic neurotransmitter receptor activity	0.007839	NPTX2/CACNG4/NPTXR/CNIH2/SHISA7/DLGAP3/DLGAP2/DLGAP4/AKAP9/HOMER1
GO:0097091	synaptic vesicle clustering	0.012461	SYN3/SYN1/CDH2/BRSK2/BRSK1/NLGN2/PCLO/RAB3A/SYNDIG1/SYN2
GO:0060080	inhibitory postsynaptic potential	0.022009	NPAS4/NLGN3/GRIK2/NLGN2/CHRNA4/INSYN1/ABAT/RIMS1/UNC13B
GO:0048172	regulation of short-term neuronal synaptic plasticity	0.028901	CLN3/SYT4/GRIK2/SHISA7/SYP/RAB3A/SHISA8
GO:0048489	synaptic vesicle transport	0.029987	KIF5A/PRKN/KIF5C/MAP2/AP3M2/AP3B2/CNIH2/LIN7A/LRRK2/PINK1/TRIM46/BLOC1S1/RAB3A/AP3S2/SNCA/AP3M1/SNAP91
GO:0098845	postsynaptic endosome	0.033863	ARC/ZDHHC2/PSD/GRIPAP1/SH3GL3/PICK1/CLSTN1/RAB4A/NSG1/STX12
GO:0097104	postsynaptic membrane assembly	0.042748	NLGN3/NLGN4X/NRXN2/NRXN1/CDH2/NLGN2
GO:0099243	extrinsic component of synaptic membrane	0.043173	CNKSR2/SCRIB/KPNA2/AKAP9/FARP1/SNAP91/RNF10/HIP1

Table S9: The astrocyte related pathways of the upregulated DEGs in cortical organoids from patient organoids with NR treatment versus non-treated patient organoids.

ID	pathway	pvalue	core_enrichment
GO:0001774	microglial cell activation	0.000443	GRN/TLR6/TLR3/CTSC/C5AR1/AIF1/PTPRC/TLR2/ITGAM/ITGB2/CCL3/C1QA
GO:0061900	glial cell activation	0.001142	GRN/TLR6/TLR3/CTSC/C5AR1/AIF1/PTPRC/TLR2/ITGAM/ITGB2/CCL3/C1QA
GO:0048712	negative regulation of astrocyte differentiation	0.029136	GPR37L1/HES5/NR2E1/ID4/KDM4A/NF1/NTRK3
GO:0045686	negative regulation of glial cell differentiation	0.035041	GPR37L1/HES5/NKX6-1/NR2E1/ID4/DLX1/DUSP10/NKX6-2/KDM4A/NF1/NTRK3
GO:0014002	astrocyte development	0.037949	LAMB2/GRN/ROR1/TLR4/C5AR1/ROR2/LAMC3/S100A8/S100A9/C1QA
GO:0021781	glial cell fate commitment	0.047561	OLIG2/HES5/ASCL1/SOX2/SOX8/SOX9/NKX2-2/PAX6/NRG1

Table S10: The neuroinflammation related pathways of the upregulated DEGs in cortical organoids from patient organoids with NR treatment versus non-treated patient organoids.

ID	pathway	pvalue	core_enrichment
GO:0050727	regulation of inflammatory response	1.00E-10	ACE/PPARA/BST1/SIGLEC10/NUPR1/SIRPA/C1QTNF12/CEBPB/PTGIS/NMI/HLA-E/GRN/RHBDF2/IL16/ADAMTS12/TRADD/TLR7/TNFRSF1A/HLA-DRB1/AOAH/PIK3CG/LRRC19/LYN/NT5E/OSMR/ZFP36/MDK/AGTR1/NEAT1/IFI35/MAPK13/MGST2/TNFRSF11A/TRPV4/CEBPA/KLF4/TNFAIP3/TEK/IGF1/GPER1/TLR3/LACC1/DUOXA2/IL6ST/GPSM3/NR1H4/DUOXA1/PTGER4/CASP1/CTSC/TLR4/PIK3AP1/NFKBIZ/PTGS2/PLCG2/SOCS3/PYCARD/CCN4/SMPDL3B/ETS1/PPARG/FOXF1/ALOX5/ANXA1/SYK/PTGES/LGALS1/SERPINF1/PTPRC/APOA1/CREB3L3/ALOX5AP/TLR2/VAMP8/STING1/ACP5/AHSG/TNFRSF1B/CD200R1/GATA3/NCF1/HCK/HGF/IL1R1/PLA2G2A/BIRC3/CASP4/S100A8/MMP9/SLAMF8/S100A9/CCL3/CD28/C3/PLA2G7/KRT1
GO:0050729	positive regulation of inflammatory response	3.25E-06	ACE/NUPR1/CEBPB/NMI/HLA-E/GRN/IL16/TRADD/TLR7/TNFRSF1A/PIK3CG/OSMR/MDK/AGTR1/NEAT1/IFI35/MAPK13/MGST2/TNFRSF11A/TRPV4/CEBPA/TLR3/IL6ST/GPSM3/PTGER4/CASP1/CTSC/TLR4/NFKBIZ/PTGS2/PLCG2/PYCARD/CCN4/ETS1/LGALS1/CREB3L3/ALOX5AP/TLR2/VAMP8/PLA2G2A/S100A8/S100A9/CCL3/CD28/C3/PLA2G7
GO:0150076	neuroinflammatory response	0.000102	TLR6/IGF1/TLR3/CTSC/C5AR1/PTGS2/PLCG2/AIF1/PTPRC/TLR2/TNFRSF1B/CD200R1/ITGAM/ITGB2/MMP9/CCL3/C1QA
GO:0002526	acute inflammatory response	0.00014	TFRC/ANO6/NUPR1/KL/CEBPB/HLA-E/HP/F3/PIK3CG/PLSCR1/OSMR/TNFRSF11A/SERPINA3/IL6ST/ASS1/TREM1/B4GALT1/IL6R/PTGS2/A2M/FN1/PTGES/CREB3L3/ALOX5AP/AHSG/GATA3/APOA2/SERPINA1/S100A8/C3/CD163
GO:0002532	production of molecular mediator involved in inflammatory response	0.001417	PLD3/APOD/PDCD4/PLA2G10/MYD88/ADCY7/PLA2G3/TICAM1/PPARA/ZC3H12A/SIRPA/SNAP23/GRN/NOS2/LYN/TLR6/GPSM3/IL4R/TLR4/PYCARD/ALOX5/SYK/CLEC7A/ALOX5AP/VAMP8/NCF1/H19/SLAMF8
GO:0002269	leukocyte activation involved in inflammatory response	0.001502	LDLR/MYD88/TRAF3IP2/GRN/SCNN1B/TLR6/TLR3/CTSC/C5AR1/AIF1/PTPRC/TLR2/ITGAM/ITGB2/CCL3/C1QA
GO:0150077	regulation of neuroinflammatory response	0.009548	NUPR1/GRN/IGF1/CTSC/PTGS2/PLCG2/PTPRC/TNFRSF1B/CD200R1/MMP9/CCL3
GO:0002437	inflammatory response to antigenic stimulus	0.010675	HLA-E/RHBDF2/HLA-DRB1/LYN/A2M/CD68/SYK/GATA3/HCK/CD28/C3
GO:0090594	inflammatory response to wounding	0.010847	SIGLEC10/HMOX1/GRN/TGFB1/MDK/TIMP1/TLR4/PPARG/ALOX5
GO:0002523	leukocyte migration involved in inflammatory response	0.011811	ADAM8/NINJ1/MDK/ALOX5/TRIM55/S100A8/SLAMF8/S100A9
GO:0061702	inflammasome complex	0.013736	NLRC4/GSDMD/CASP1/PYCARD/CASP4
GO:0002673	regulation of acute inflammatory response	0.014727	HLA-E/PIK3CG/OSMR/TNFRSF11A/IL6ST/PTGS2/PTGES/CREB3L3/ALOX5AP/C3
GO:0002861	regulation of inflammatory response to antigenic stimulus	0.015639	HLA-E/RHBDF2/HLA-DRB1/LYN/SYK/HCK/CD28/C3
GO:0002675	positive regulation of acute inflammatory response	0.019835	HLA-E/PIK3CG/OSMR/TNFRSF11A/IL6ST/PTGS2/CREB3L3/ALOX5AP/C3
GO:0002534	cytokine production involved in inflammatory response	0.044761	PLD3/APOD/PDCD4/PLA2G10/MYD88/ADCY7/PLA2G3/TICAM1/PPARA/ZC3H12A/SIRPA/NOS2/TLR6/GPSM3/TLR4/PYCARD/ALOX5/CLEC7A/H19
GO:1900015	regulation of cytokine production involved in inflammatory response	0.044761	PLD3/APOD/PDCD4/PLA2G10/MYD88/ADCY7/PLA2G3/TICAM1/PPARA/ZC3H12A/SIRPA/NOS2/TLR6/GPSM3/TLR4/PYCARD/ALOX5/CLEC7A/H19

Table S11. Primary antibodies using in immunostainings.

**Primary Antibodies.**				
**Name**	Supplier	Species	Catalogue Number	Concentration
**Anti-NDUFB10**	Abcam	Rabbit	Ab196019	1:300
**MAP2 Antibody (Conjugated PE)**	Santacruz Biotechnology	Mouse	Sc-74421	1:200
**Anti-GFAP Antibody**	Abcam	Chicken	Ab4674	1:200
**Anti-mtTFA Antibody (Conjugated Alexa flour® 488)**	Abcam	Mouse	Ab198308	1:200
**VDAC1/Porin Antibody (Conjugated Alexa flour® 647)**	Santacruz Biotechnology	Mouse	Sc-390996	1:200
**Anti-SOX2 Antibody**	Abcam	Rabbit	Ab97959	1:100
**Anti-MAP2 Antibody**	Abcam	Chicken	Ab5392	1:100
**Recombinant Anti-GFAP antibody**	Abcam	Mouse	Ab279289	1:500
**Anti-Oct 4 Antibody**	Abcam		ab19857	1:100
**Anti-SSEA4 Antibody**	Abcam		ab16287	1:200
**Anti-Nestin Antibody**	Santa Cruz Biotechnology		sc23927	1:50
**Anti-NeuN Antibody**	Cell Signaling		24307	1:500
**Anti-Tuj1 Antibody**	Abcam		ab78078	1:1000
**Anti-SATB2 Antibody**	Abcam	Rabbit	ab4674	1:400
**Anti-Tuj1 Antibody**	Abcam	Rat	ab18465	1:500

Table S12. Secondary antibodies using in immunostainings.

**Secondary Antibodies.**			
**Name**	Supplier	Catalogue Number	Concentration
**Goat anti-Rabbit IgG (H+L), Alexa Fluor™ 488**	Thermo Fisher Scientific	A11008	1:800
**Goat anti-Chicken IgY (H+L), Alexa Fluor™ 647**	Thermo Fisher Scientific	A21449	1:800
**Goat anti-Rabbit IgG (H+L), Alexa Fluor™ 594**	Thermo Fisher Scientific	A11012	1:800
**Goat anti-Mouse IgG2b, Alexa Fluor™ 488**	Thermo Fisher Scientific	A21141	1:800
**Goat anti-Chicken IgY (H+L) Secondary Antibody, Alexa Fluor™ 594**	Thermo Fisher Scientific	A11042	1:800
**Goat anti-Mouse IgG (H+L) Highly Cross-Adsorbed Secondary Antibody, Alexa Fluor™ 647**	Thermo Fisher Scientific	A21236	1:800

Table S13: The numbers of the clones and replications in the Figure 1, 2.

Numbers of the clones and replications					
**Figure**	Detroit 551 (clone 1/clone2/clone3)	CRL2097	AG05836	Patient 1 (clone 1)	Patient 1 (clone 2)
**1E**	5/5/5	5	5	8	10
**1F**	5/5/5	5	5	8	10
**1G**	5/5/5	5	5	8	10
**1H**	5/5/3	3	4	8	10
**1I**	4/4/4	4	4	8	10
**1J**	4/4/4	4	4	8	10
**1K**	4/4/4	4	4	8	10
**1L**	0/4/4	10	0	6	6
**1M**	0/3/0	5	0	3	3
**1N**	0/3/3	3	0	4	3
**1O**	0/3/3	3	0	4	3
**1P**	0/2/2	4	0	4	3
**1Q**	0/2/2	4	0	4	3
**1R**	0/3/3	3	0	4	3
**2E**	6/3/5	4	4	6	3
**2F**	6/3/5	4	4	6	3
**2G**	6/3/5	4	4	6	3
**2H**	6/6/9	8	10	6	6
**2I**	2/2/2	3	2	6	6
**2J**	3/3/3	3	3	3	3
**2L**	1/3/3	3	0	3	6
**2M**	0/2/2	3	0	3	5
**2N**	3/3/5	3	0	3	3
**2O**	4/6/3	10	0	6	6
**2P**	0/4/4	3	0	5	5
**2Q**	0/3/3	8	0	3	M
**2R**	4/4/3	4	0	3	3
**2S**	0/4/6	3	0	3	3
**2T**	0/3/0	3	0	5	5
**2U**	0/4/6	3	0	3	3
**2V**	0/3/0	3	0	5	5

Table S14: The numbers of the clones and replications in the Figure 5, 7.

**Numbers of the clones and replications**					
**Figure**	Detroit 551 (clone 1/clone2/clone3)	CRL2097	AG05836	Patient 1 (clone 1)	Patient 1 (clone 2)
**5D**	0/2/0	2	0	2	2
**5E**	0/2/0	2	0	2	2
**5F**	0/2/0	2	0	2	2
**5H**	1/2/0	0	0	3	0
**5I**	1/2/0	0	0	3	0
**5G**	1/2/0	0	0	3	0
**5L**	1/2/0	3	0	7	8
**5M**	0/3/0	5	0	6	6
**7D**	0/2/0	2	0	2	2
**7E**	0/2/0	2	0	2	3
**7F**	0/2/0	2	0	2	3
**7H**	0/2/0	2	0	2	3
**7I**	0/2/0	2	0	2	3
**7J**	0/2/0	2	0	2	3
**7L**	5/5/0	5	0	5	5
**7M**	3/3/3	4	0	3	3

Table S15: The numbers of the clones and replications in the supplementary Figures.

**Numbers of the clones and replications**						
**Figure**	Detroit 551 (clone 1/clone2/clone 3)	CRL2097	AG05836	Patient 1 (clone 1)	Patient 1 (clone 2)	Patient 2
**Supplementary Figure 3**	3/3/0	3	2	0	3	0
**Supplementary Figure 4**	0/0/0	0	0	3	0	0
**Supplementary Figure 5A**	3/3/0	3	0	4	4	0
**Supplementary Figure 5B**	5/4/3	3	4	9	10	0
**Supplementary Figure 5C**	0/3/0	3	0	3	3	0
**Supplementary Figure 13**	0/0/0	3	0	0	0	3
**Supplementary Figure 14**	0/0/0	3	0	0	0	3
**Supplementary Figure 16**	0/0/0	3	0	0	0	3

Supplemental figures

NANOG

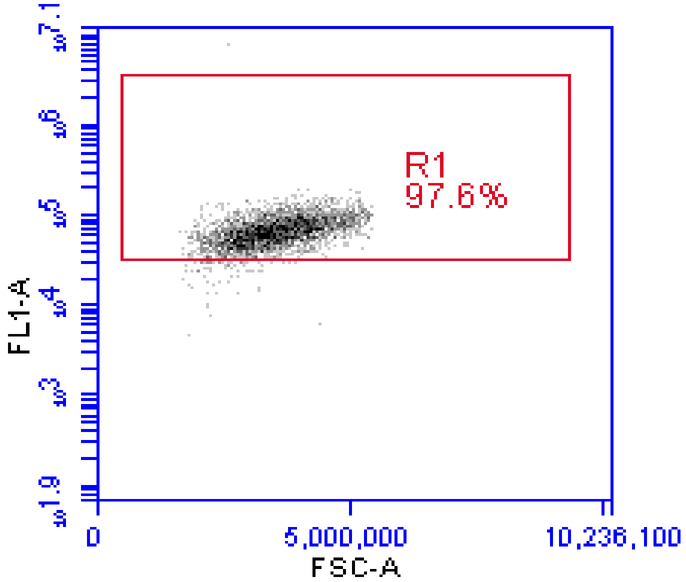

Supplementary Figure 1: Flow cytometry analysis of Nanog-positive cells.

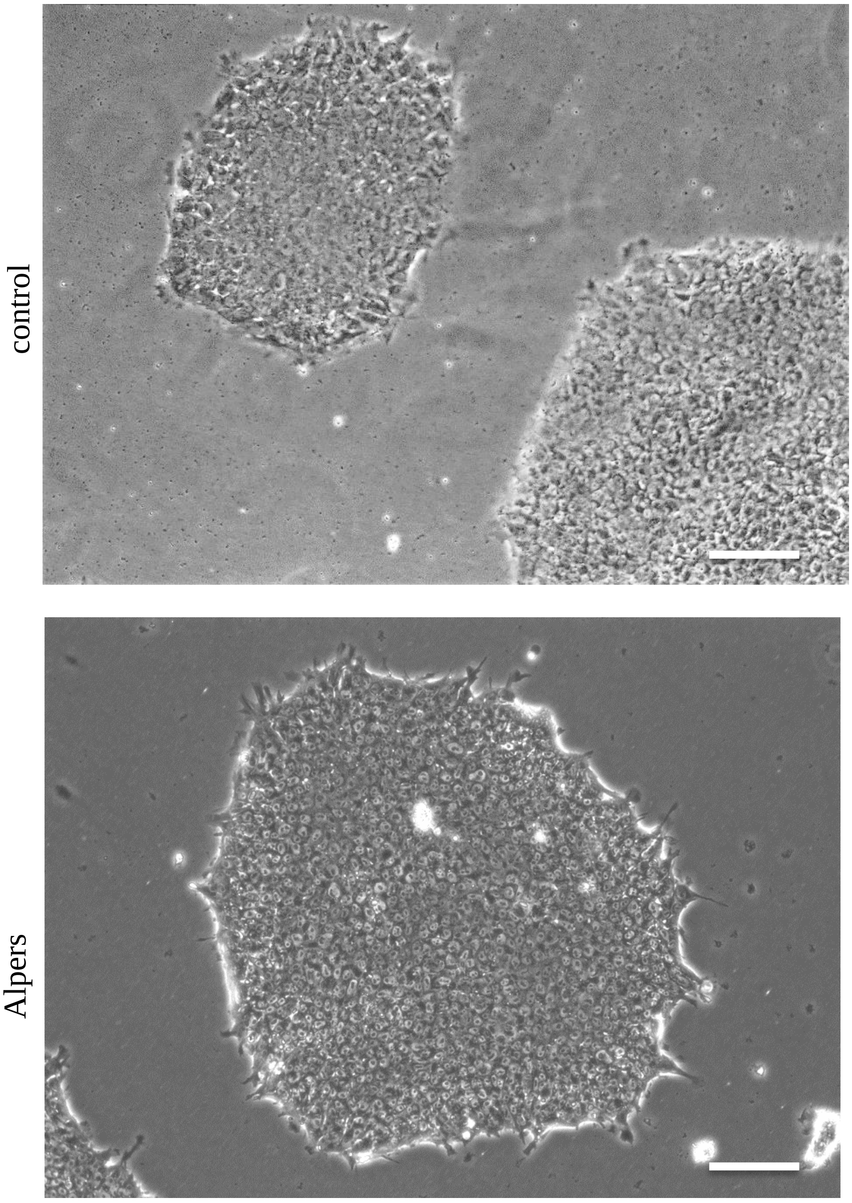

Supplementary Figure 2: Phase-contrast images in control and Alpers' iPSCs. Scale bar is 100 µm.

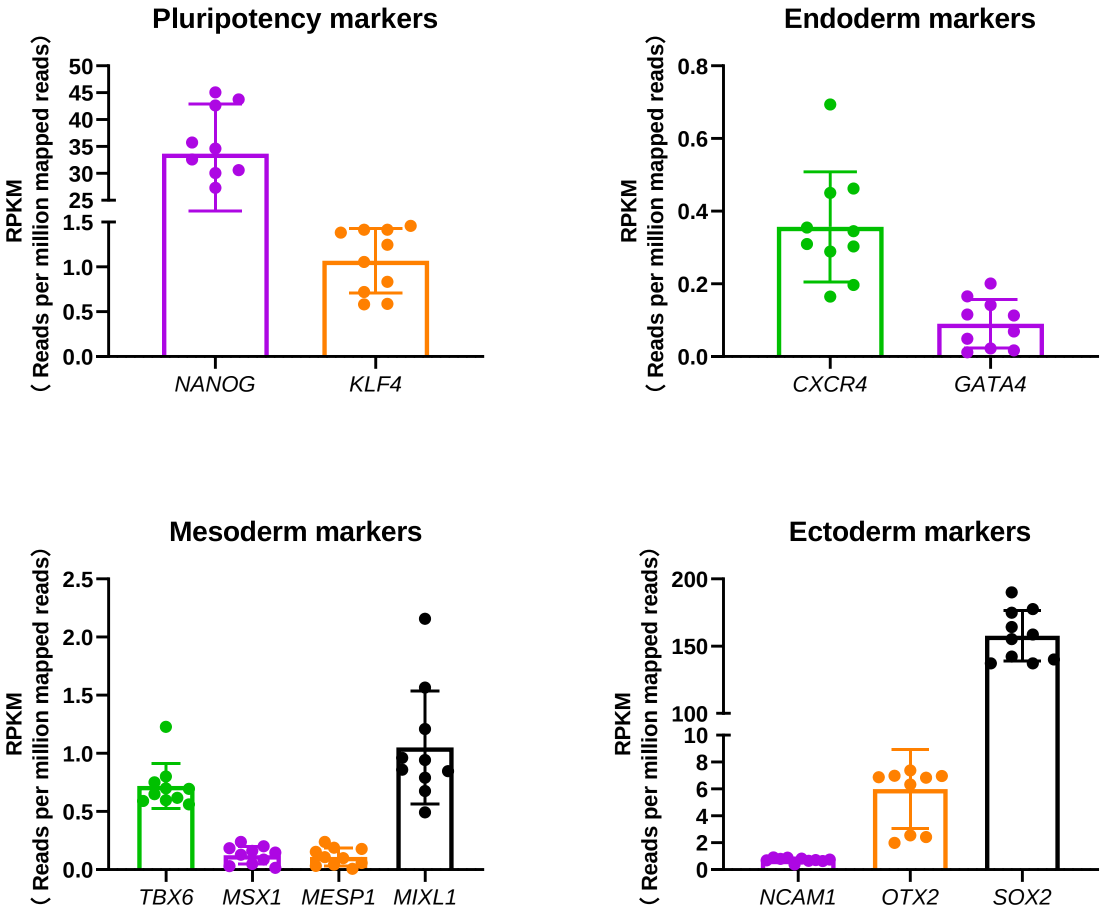

Supplementary Figure 3: Gene expression of the pluripotent, endoderm, mesoderm and ectoderm makers in control iPSCs.

The numbers of clones and replications in each experiment were listed in Table S 15.

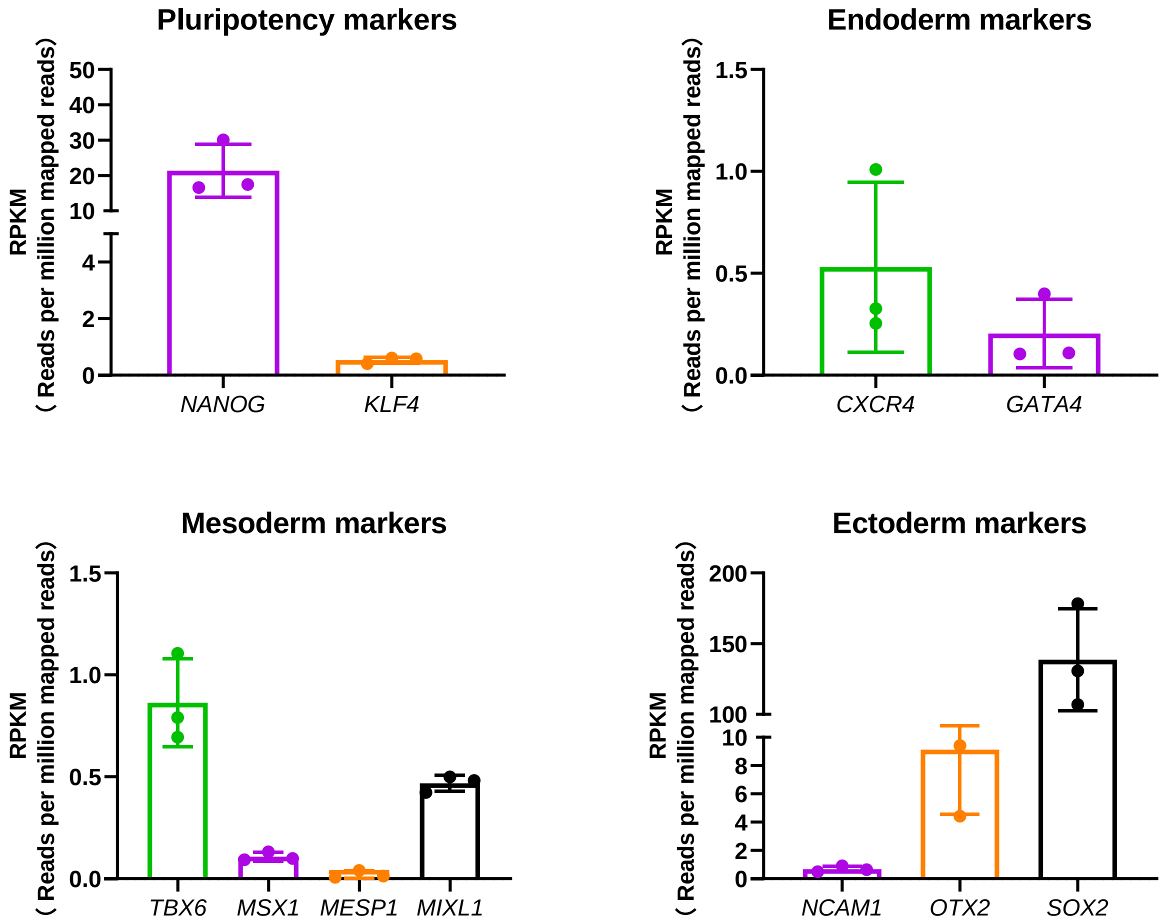

Supplementary Figure 4: Gene expression of the pluripotent, endoderm, mesoderm and ectoderm makers in Alpers' iPSCs.

The numbers of clones and replications in each experiment were listed in Table S 15.

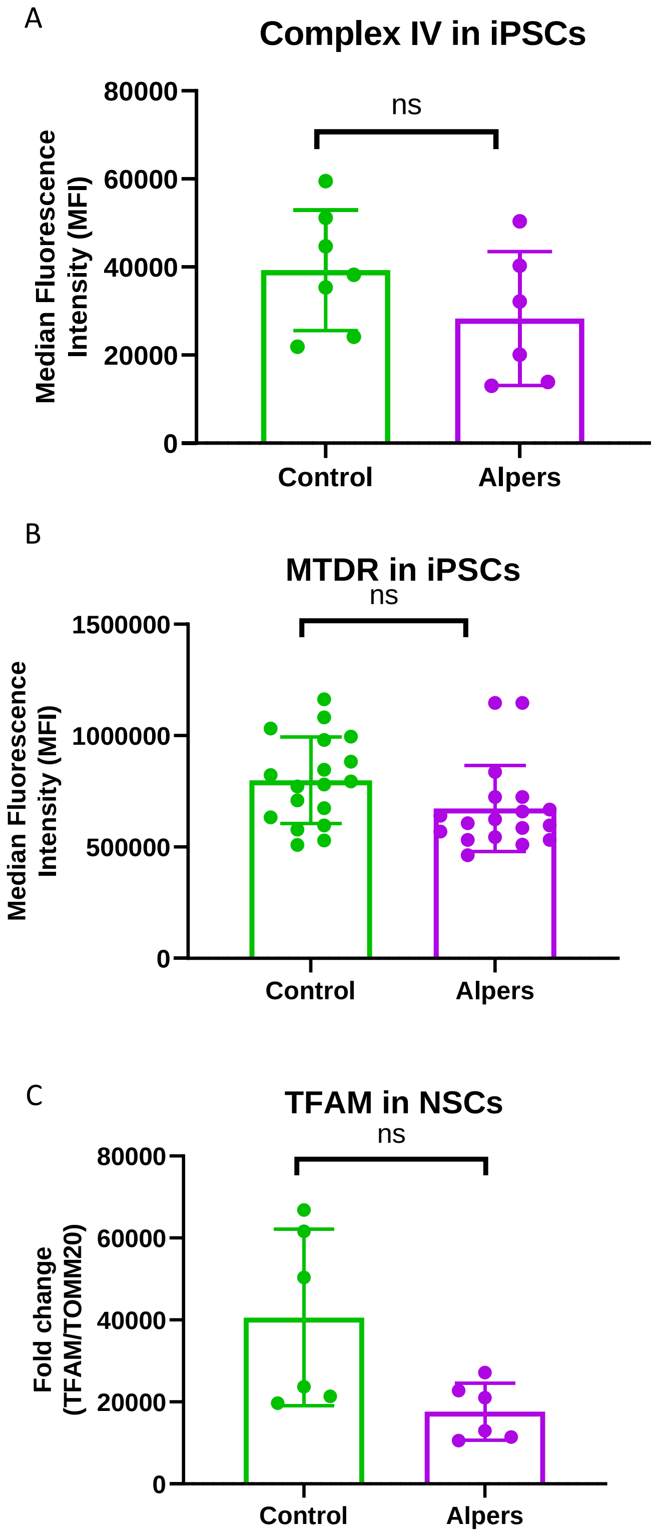

Supplementary Figure 5: Flow cytometric analysis of CIV levels and MTDR in iPSCs, and TFAM expression in NSCs.

The numbers of clones and replications in each experiment were listed in Table S 15.

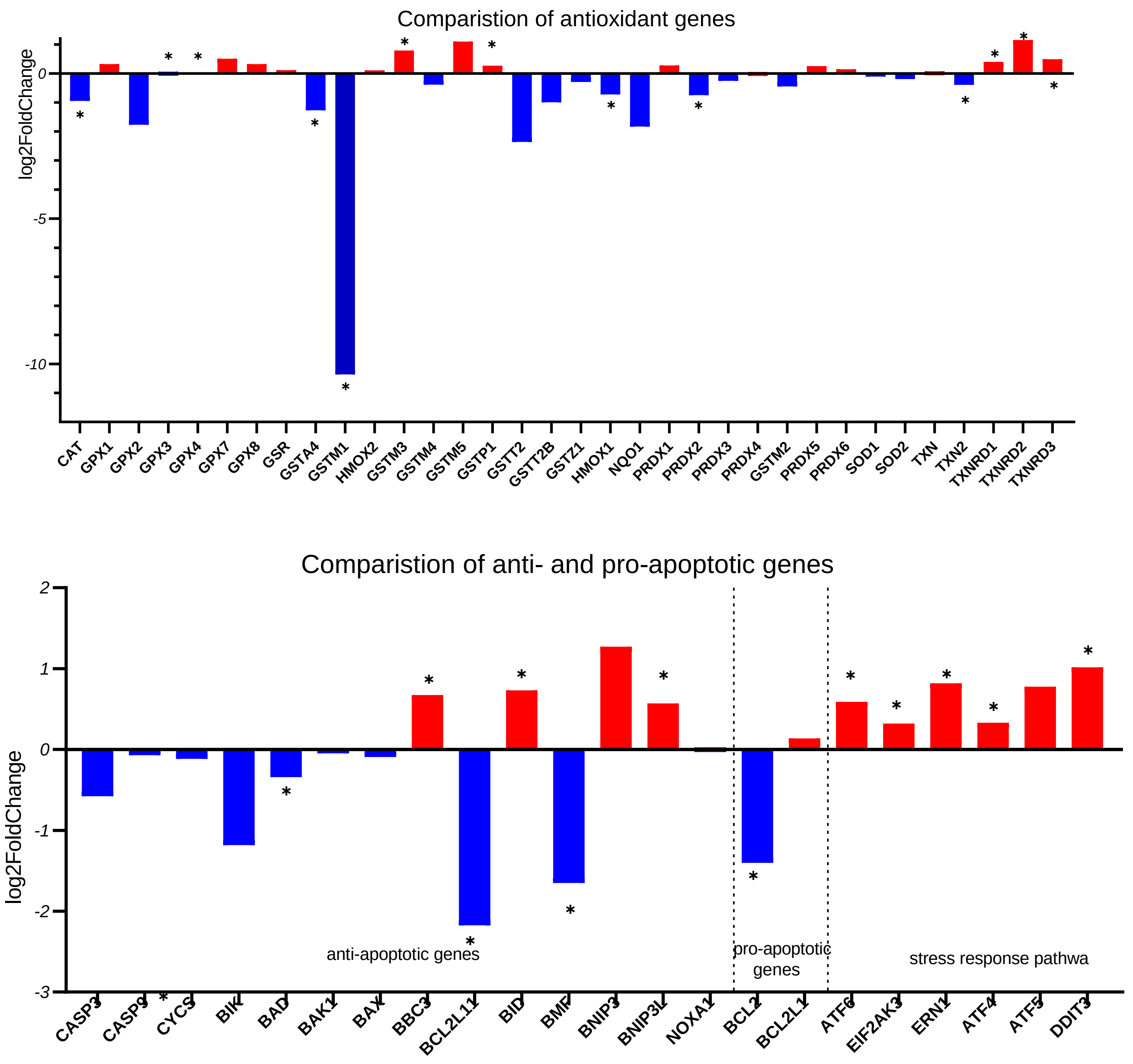

Supplementary Figure 6: The comparison of antioxidant genes (up), anti- and pro-apoptotic genes (down) in Alpers' NSCs comparing to the control NSCs. The vertical axis is labeled as "log2 Fold change,", each bar represents the fold change of a gene, with bars above the horizontal axis indicating increased expression (upregulation) and bars below indicating decreased expression (downregulation). The asterisks likely indicate that the change in expression for those genes is statistically significant.

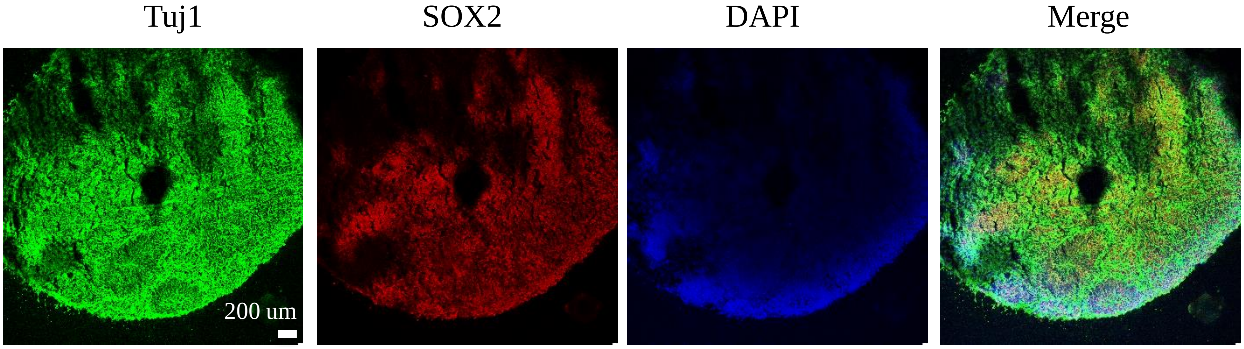

Supplementary Figure 7: Fluorescent staining of cortical organoid section using neuron marker Tuj1 and neural progenitor marker SOX2 at day 40 in control line. Nuclei are stained with DAPI (blue). Scale bar is 200 µm.

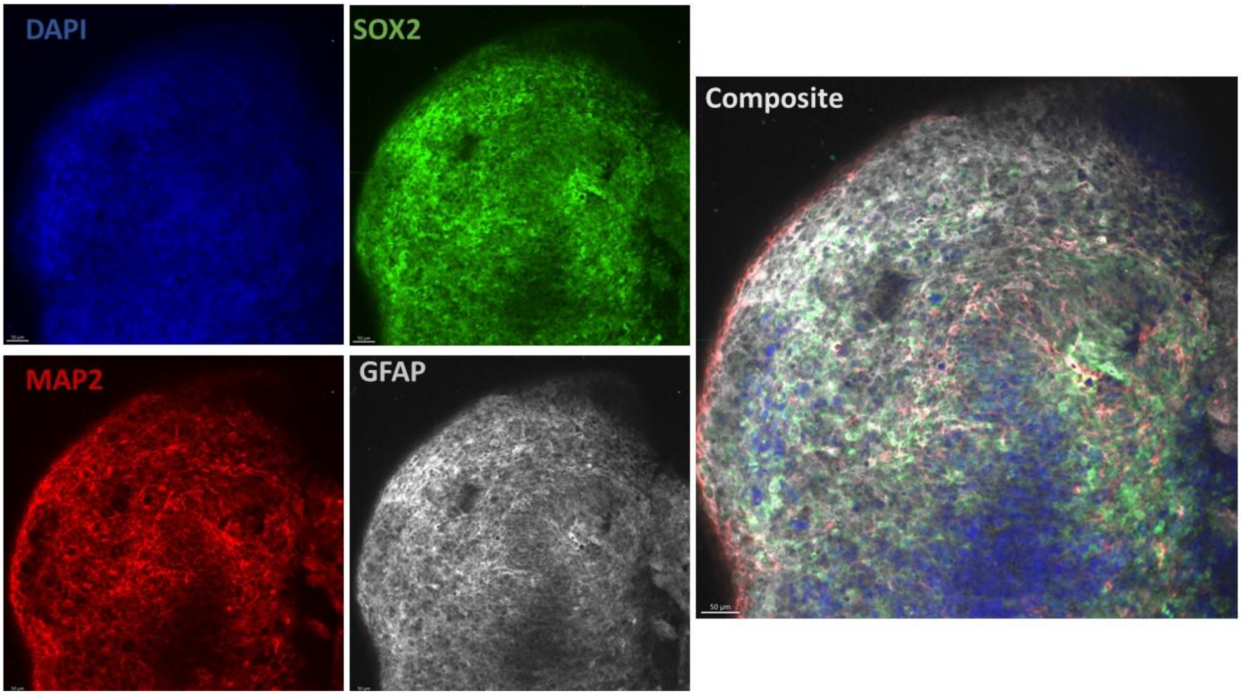

Supplementary Figure 8: Fluorescent staining of cortical organoid section using neuron marker MAP2 (red), astrocyte marker GFAP (white), and neuron progenitor marker SOX2 (green) in Alpers' line (clone 1). Nuclei are stained with DAPI (blue). Scale bar is 50 µm.

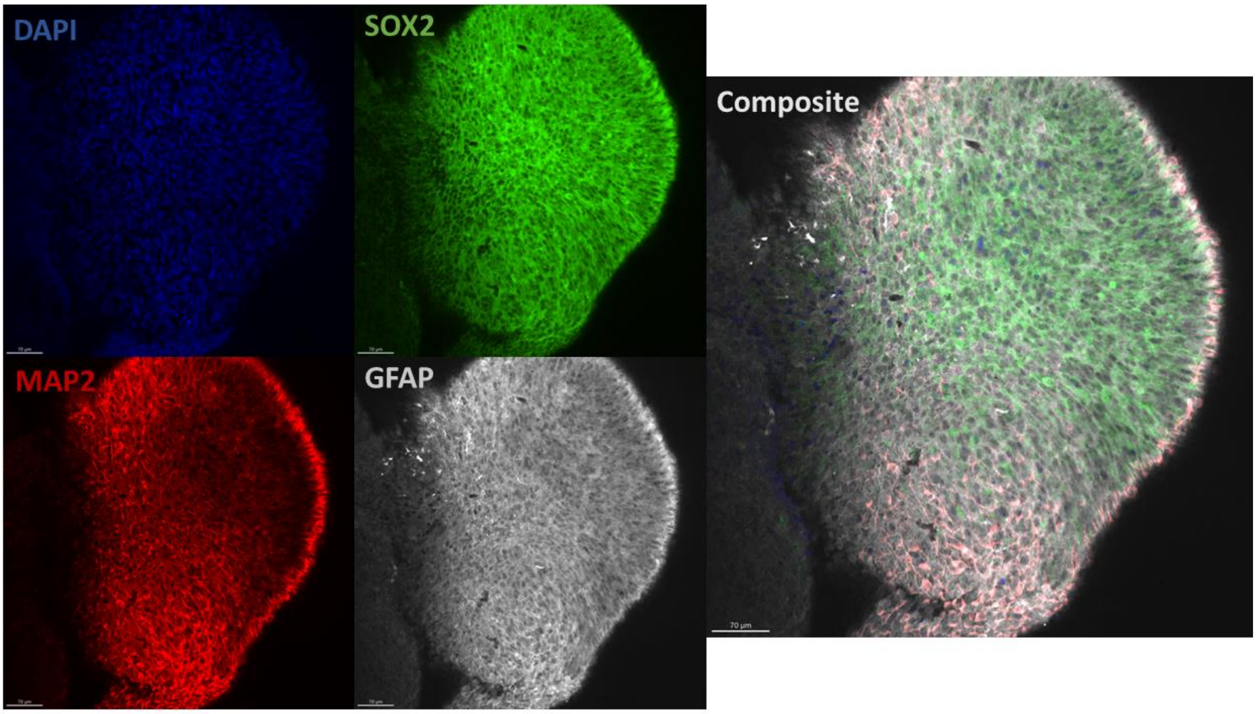

Supplementary Figure 9: Fluorescent staining of cortical organoid section using neuron marker MAP2 (red), astrocyte marker GFAP (white), and neuron progenitor marker SOX2 (green) in Alpers' line (clone 2). Nuclei are stained with DAPI (blue). Scale bar is 70 µm.

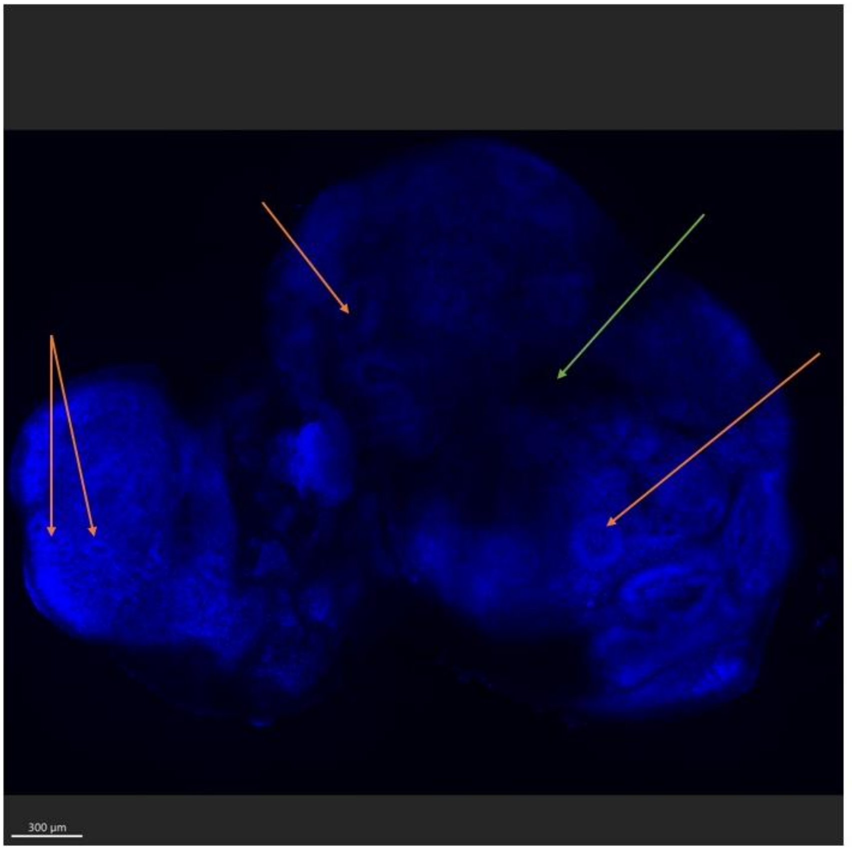

Supplementary Figure 10: Representative image of deep layer of organoid with DAPI stain in Alpers' line (clone 1). Orange arrows point to circular configuration of nuclei reminiscent of neural rosette structures. Green arrow shows area devoid of DAPI assumed to be either apoptotic or left unstained due to limited permeability. Scale bar is 300 µm.

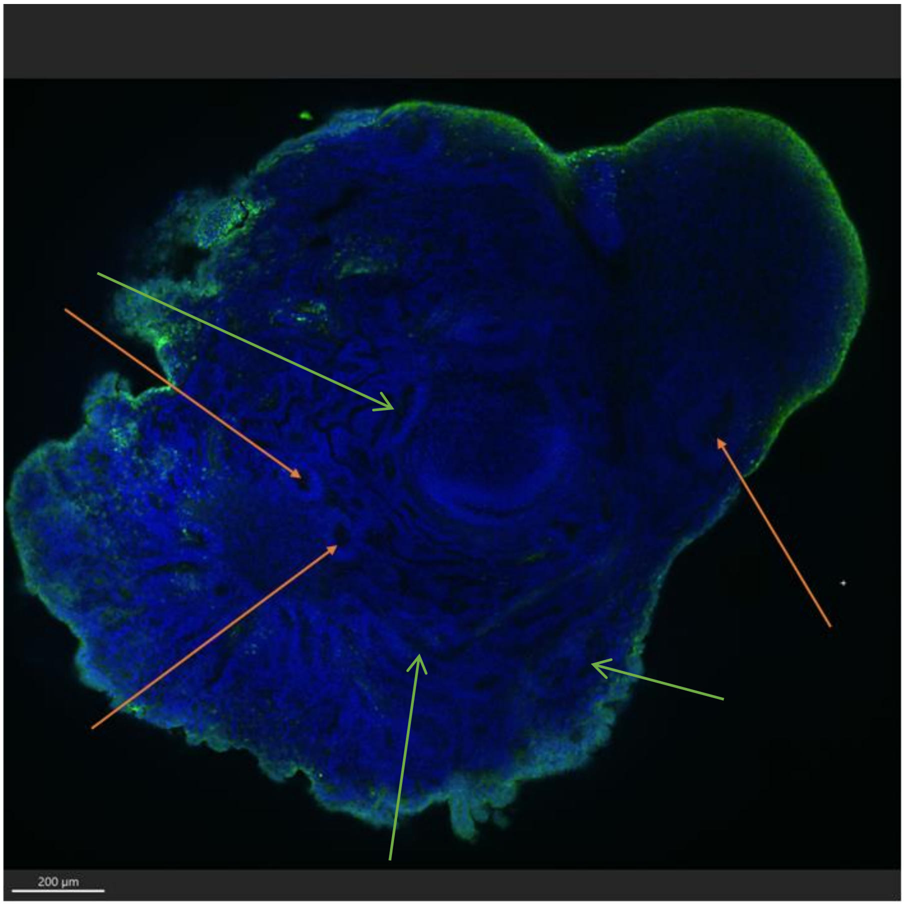

Supplementary Figure 11: Representative image of deep layer of organoid with DAPI stain in Alpers' line (clone 2). Orange arrows point to circular configuration of nuclei reminiscent of neural rosette structures. Green arrow shows area devoid of DAPI assumed to be either apoptotic or left unstained due to limited permeability. Scale bar is 200 μm.

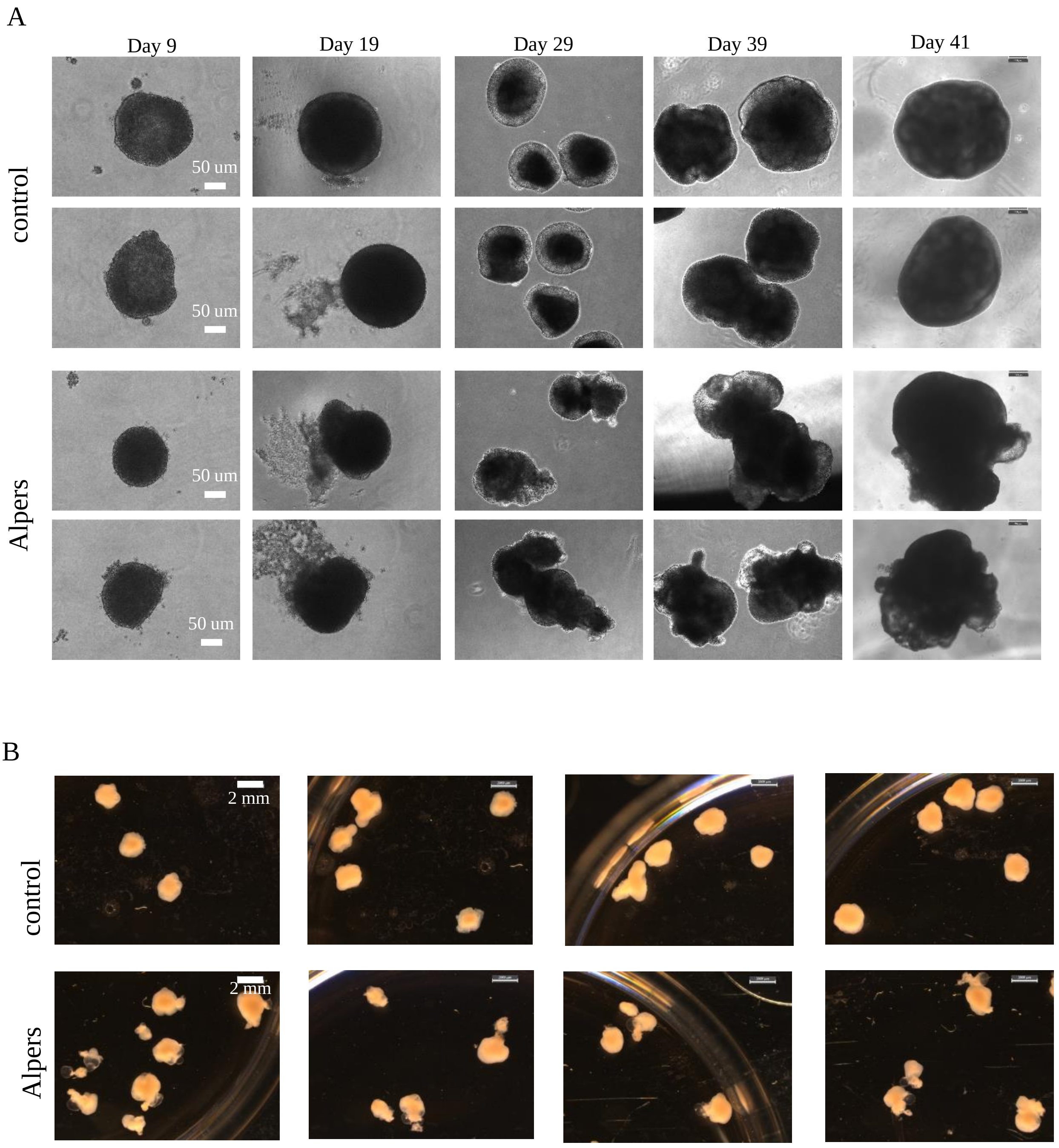

Supplementary Figure 12: Phase-contrast images (A) and bright field images (B)of cortical organoids of control and Alpers' patient line during the differentiation (A) and at day 37 (B). Scale bar is 100 µm or 2 mm.

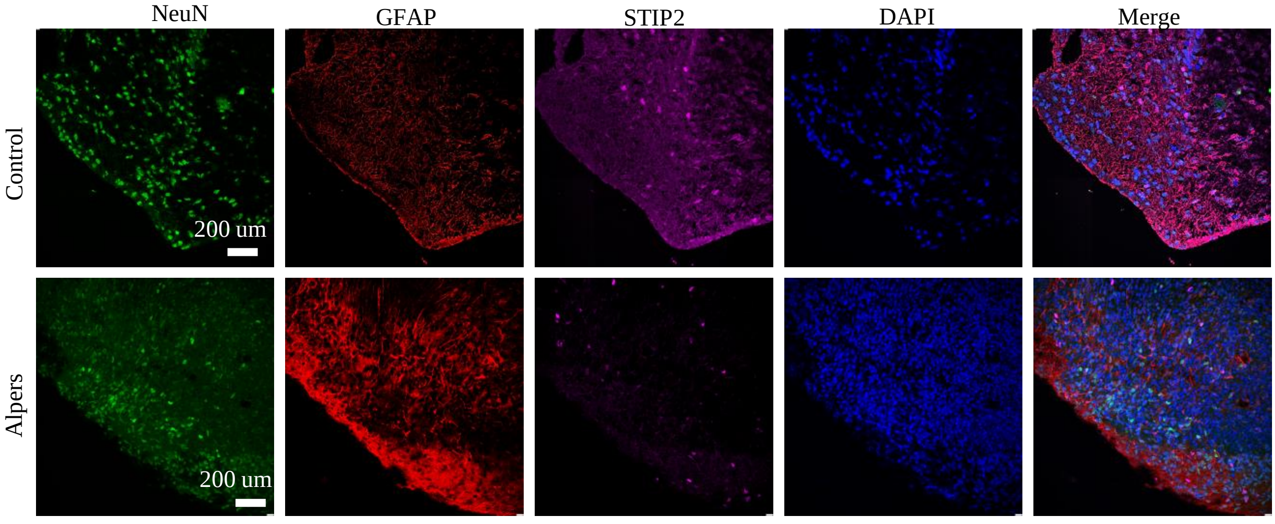

Supplementary Figure 13: Fluorescent staining of cortical organoid section using neuron marker NeuN, astrocyte marker GFAP, and cortical neuron marker MAP2 at day 30 in control and Alpers' line. Nuclei are stained with DAPI (blue). Scale bar is 200 µm.

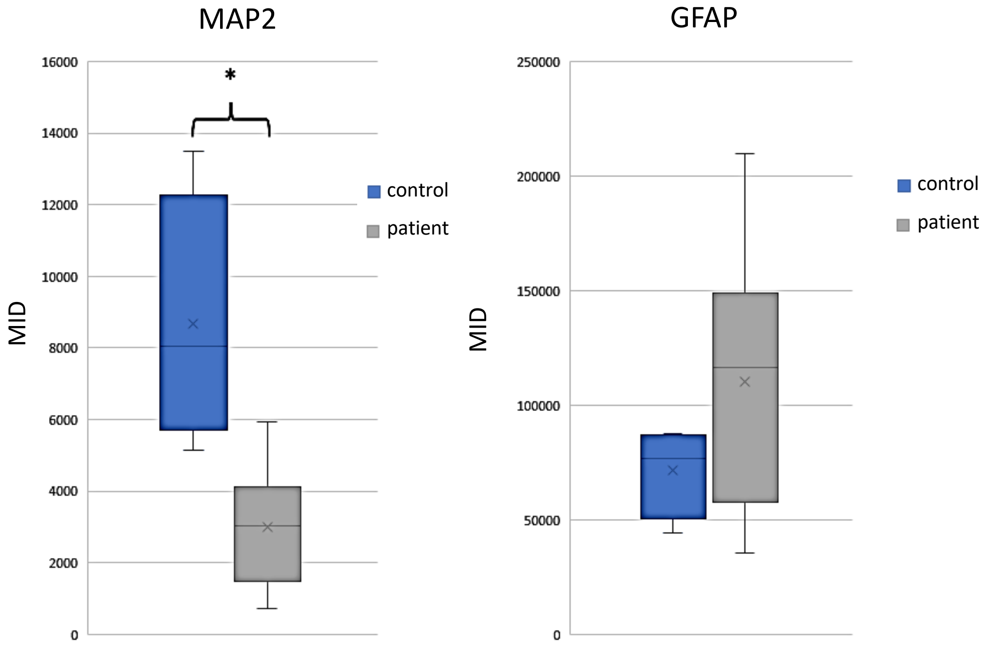

Supplementary Figure 14: Quantification of the immortalizing showing mean of integrated density (MID) values for marker MAP2, and GFAP. Values are obtained from measuring different regions of an individual organoids of each line. Boxes show median values and upper and lower quartiles. X indicates the mean value. Whiskers show the highest and lowest values. Dots indicate outlier values. Fluorescent analysis was performed in ImageJ using the threshold and measure function. The data was represented as mean +/- SD, $n \geq 4$. Significant values ($P \leq .05$) are annotated with (*).

The numbers of clones and replications in each experiment were listed in Table S 15.

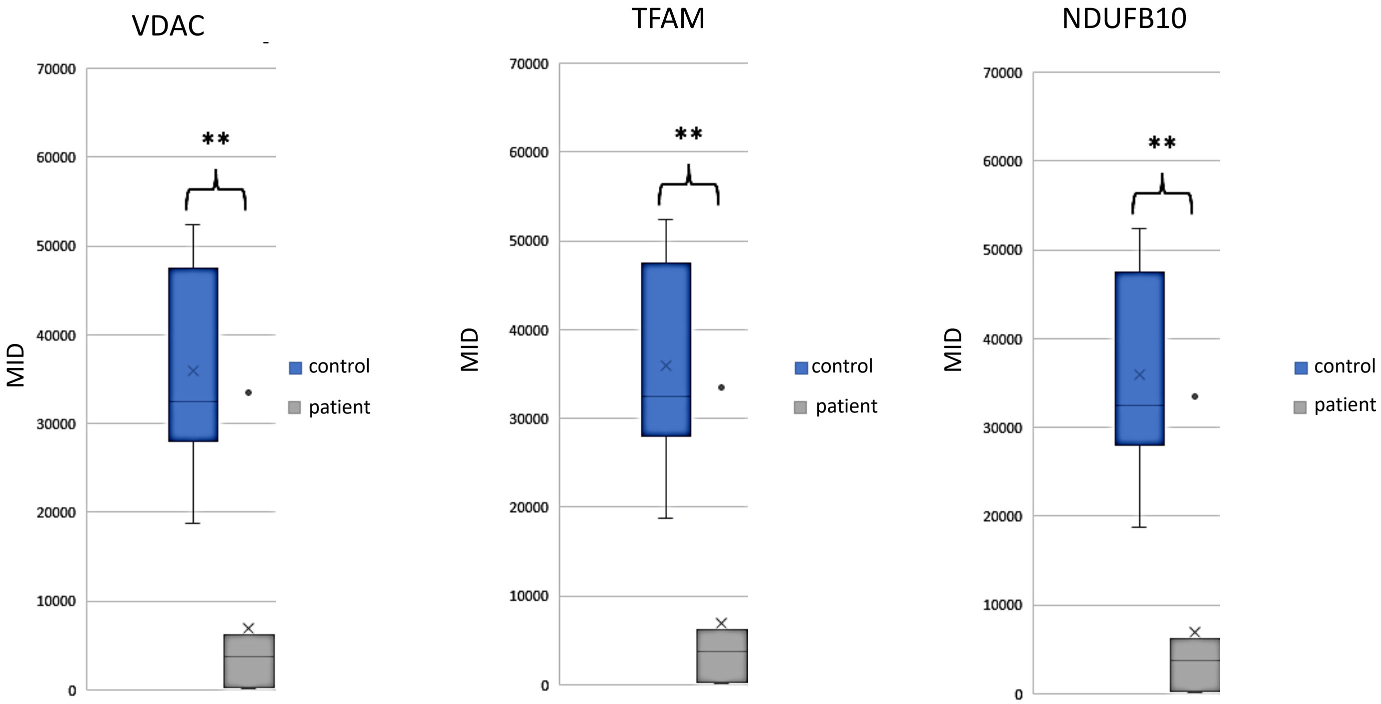

Supplementary Figure 15: Quantification of the immortalizing showing mean of integrated density (MID) values of patient and control cortical organoids for marker VDAC, TFAM and NDUFB10. Values are obtained from measuring different regions of an individual organoid of each line. Boxes show median values and upper and lower quartiles. X indicates the mean value. Whiskers show the highest and lowest values. Dots indicate outlier values. Fluorescent Analysis was performed in ImageJ using the threshold and measure function. The data was represented as mean +/- SD, $n \geq 5$. Highly Significant values ($P \leq .01$) are annotated with (**).

The numbers of clones and replications in each experiment were listed in Table S 15.

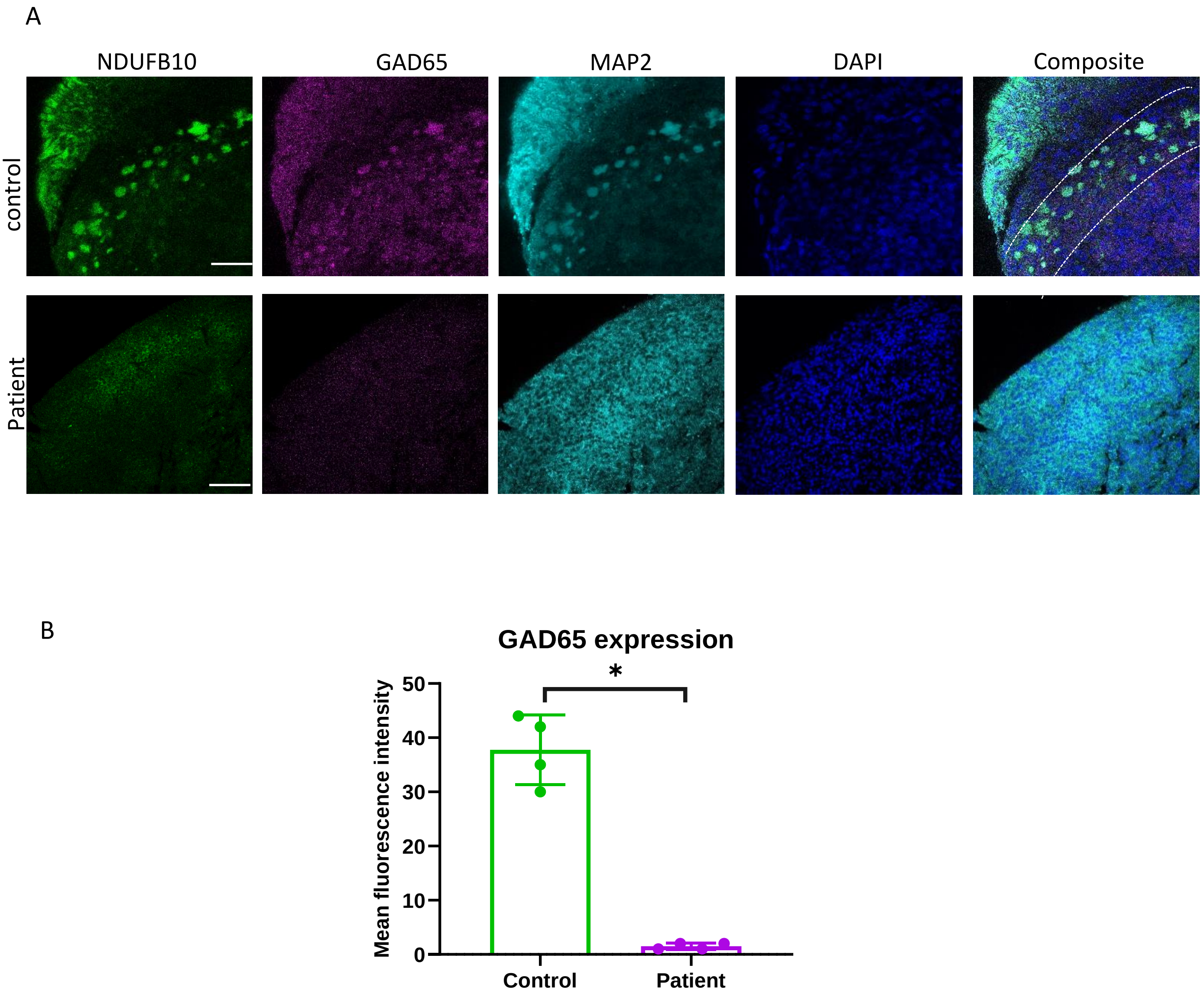

Supplementary Figure 16: Fluorescent staining (A) and quantification of the fluorescent intensity (B) for cortical organoid section using interneuron marker GAD 65 (purple), neuron marker MAP2 (cyan) and complex I subunit NDUFB10 (green) in control and Alpers' line. Nuclei are stained with DAPI (blue). Scale bar is 50 µm.

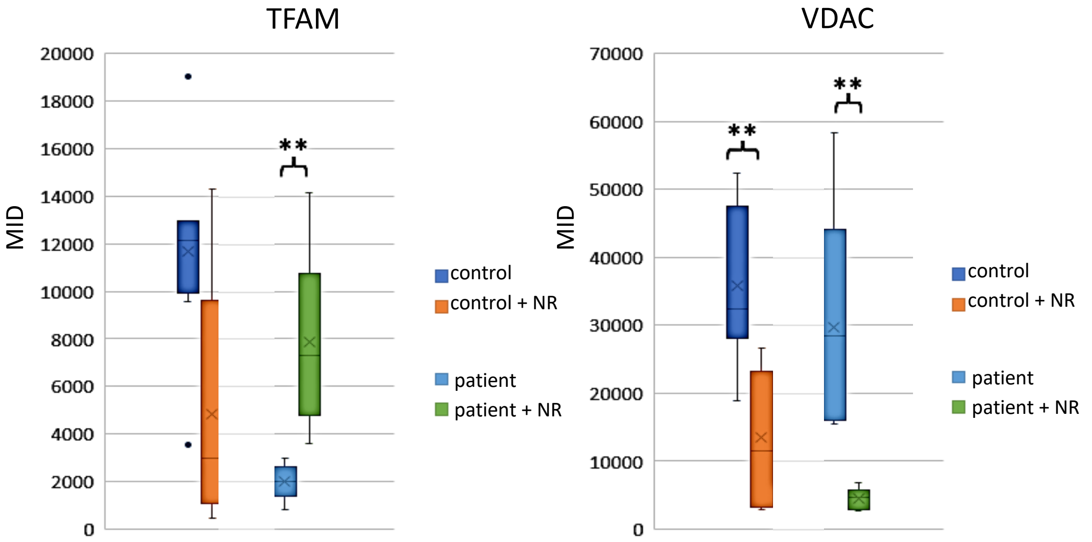

Supplementary Figure 17: Quantification of the fluorescent intensity TFAM and VDAC for control and patient cortical organoid before and after NR treatment. Values are obtained from measuring different regions of an individual organoids of each line. Boxes show median values and upper and lower quartiles. X indicates the mean value. Whiskers show the highest and lowest values. Dots indicate outlier values. Fluorescent Analysis was performed in ImageJ using the threshold and measure function. The data was represented as mean +/- SD, n≥ 5. Highly significant differences ($P \leq .01$) are annotated with (**).

The numbers of clones and replications in each experiment were listed in Table S 15.

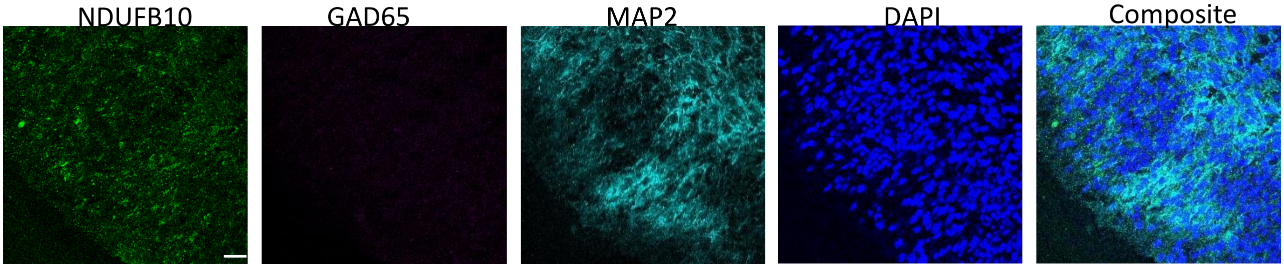

Supplementary Figure 18: Fluorescent staining of cortical organoid section using interneuron marker GAD 65 (purple), neuron marker MAP2 (cyan) and complex I subunit NDUFB10 (green) in Alpers' line in the presence of NR treatment. Nuclei are stained with DAPI (blue). Scale bar is 50 µm.

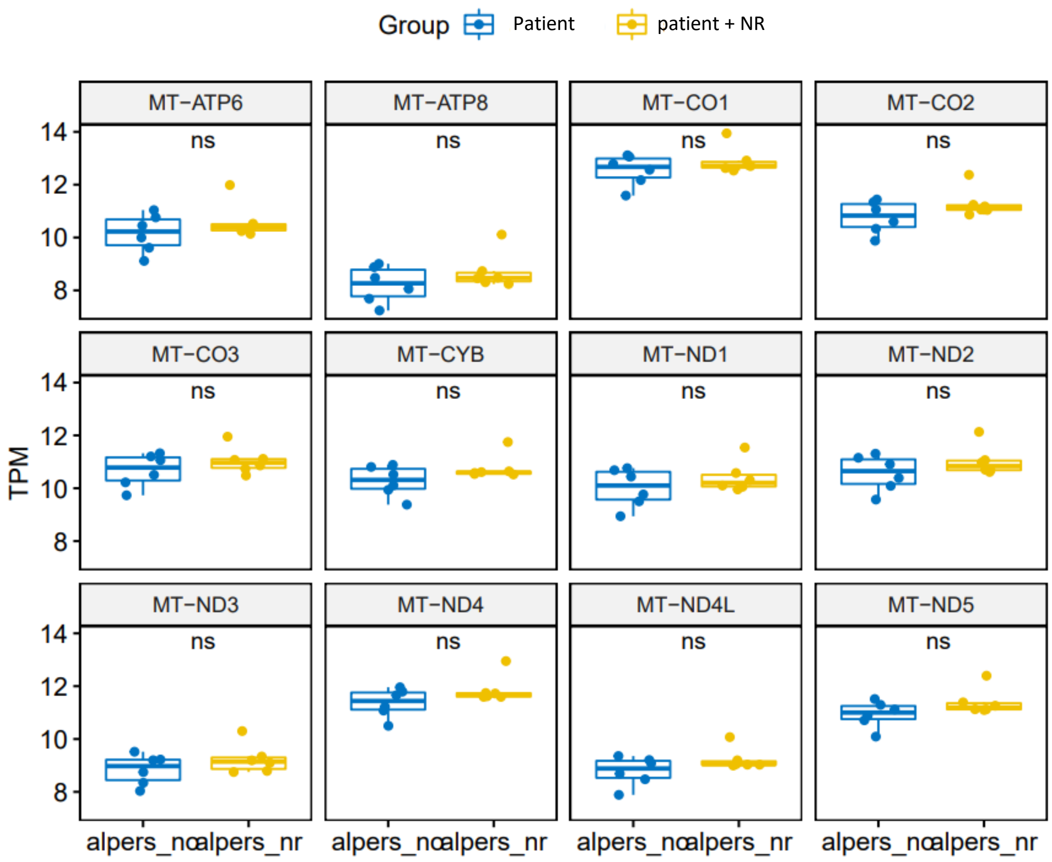

Supplementary Figure 19: RNA expressions of mitochondrial genes in Alpers' organoids before and after NR treatment.
